# Competitive Endogenous RNA Network Activates Host Immune Response in SARS-CoV-2-, panH1N1 (A/California/07/2009)-, and H7N9 (A/Shanghai/1/2013)-Infected Cells

**DOI:** 10.3390/cells11030487

**Published:** 2022-01-30

**Authors:** Minghui Yang, Jin Li, Shoulong Deng, Hao Fan, Yun Peng, Guoguo Ye, Jun Wang, Jinli Wei, Xiao Jiang, Zhixiang Xu, Ling Qing, Fuxiang Wang, Yang Yang, Yingxia Liu

**Affiliations:** 1 Shenzhen Key Laboratory of Pathogen and Immunity, National Clinical Research Center for Infectious Disease, State Key Discipline of Infectious Disease, Shenzhen Third People’s Hospital, Second Hospital Affiliated to Southern University of Science and Technology, Shenzhen 518112, China; yangmh16@cau.edu.cn (M.Y.); yun_peng403@163.com (Y.P.); yegg1916@163.com (G.Y.); wj18129973160@163.com (J.W.); y13266735776@163.com (J.W.); 13823173116@163.com (X.J.); 13823527608@163.com (Z.X.); qing2021626@163.com (L.Q.); ganransanke-01@szsy.sustech.edu.cn (F.W.); 2 School of Public Health (Shenzhen), Sun Yat-sen University, Shenzhen 518406, China; lijin66@mail2.sysu.edu.cn; 3 NHC Key Laboratory of Human Disease Comparative Medicine, Institute of Laboratory Animal Sciences, Chinese Academy of Medical Sciences and Comparative Medicine Center, Peking Union Medical College, Beijing 100021, China; dengshoulong@cnilas.org; 4 Section of Hematology and Oncology, Department of Medicine, The University of Chicago, Chicago, IL 60637, USA; haofan@uchicago.edu

**Keywords:** SARS-CoV-2, IRF1, influenza A, autophagy, noncoding RNA

## Abstract

The global outbreak of severe acute respiratory syndrome coronavirus 2 (SARS-CoV-2) is still ongoing, as is research on the molecular mechanisms underlying cellular infection by coronaviruses, with the hope of developing therapeutic agents against this pandemic. Other important respiratory viruses such as 2009 pandemic H1N1 and H7N9 avian influenza virus (AIV), influenza A viruses, are also responsible for a possible outbreak due to their respiratory susceptibility. However, the interaction of these viruses with host cells and the regulation of post-transcriptional genes remains unclear. In this study, we detected and analyzed the comparative transcriptome profiling of SARS-CoV-2, panH1N1 (A/California/07/2009), and H7N9 (A/Shanghai/1/2013) infected cells. The results showed that the commonly upregulated genes among the three groups were mainly involved in autophagy, pertussis, and tuberculosis, which indicated that autophagy plays an important role in viral pathogenicity. There are three groups of commonly downregulated genes involved in metabolic pathways. Notably, unlike panH1N1 and H7N9, SARS-CoV-2 infection can inhibit the m-TOR pathway and activate the p53 signaling pathway, which may be responsible for unique autophagy induction and cell apoptosis. Particularly, upregulated expression of IRF1 was found in SARS-CoV-2, panH1N1, and H7N9 infection. Further analysis showed SARS-CoV-2, panH1N1, and H7N9 infection-induced upregulation of lncRNA-34087.27 could serve as a competitive endogenous RNA to stabilize IRF1 mRNA by competitively binding with miR-302b-3p. This study provides new insights into the molecular mechanisms of influenza A virus and SARS-CoV-2 infection.

## 1. Introduction

Severe acute respiratory syndrome coronavirus 2 (SARS-CoV-2) was found to infect human beings in late December 2019, in Wuhan, China, and Coronavirus disease 2019 (COVID-19) caused by SARS-CoV-2 was declared a global pandemic by the World Health Organization (WHO) on 11 March 2020 [1,2]. It has since spread to the majority of countries across the world. Notably, asymptomatic infections account for 13–30.8% of all SARS-CoV-2 infections, and silent transmission during the presymptomatic and asymptomatic stages is responsible for more than 50% of the overall attack rate in COVID-19 outbreaks [3,4,5]. There are more than 127 million confirmed cases and over 2.7 million deaths thus far [6]. Approximately 16–21% of COVID-19 patients have become severely ill, of which there has been a 2–3% mortality rate [7,8,9,10]. Additionally, there were recent outbreaks of new influenza strains, such as the 2009 pandemic H1N1 (panH1N1), which emerged in North America [11], and a novel virus outbreak of H7N9 a few years later [12]. Both SARS-CoV-2 and influenza A (panH1N1, H7N9) viruses infect the respiratory tract and exhibit similar symptoms such as fever, cough, headache, chill, stuffy nose, sore throat, etc. Respiratory complications seen in patients with influenza and COVID-19 are pneumonia and, in severe cases, ARDS [13,14,15]. Moreover, they both use surface proteins to infect the host, influenza A viruses use hemagglutinin and neuraminidase, whereas SARS-CoV-2 uses S protein [16,17]. Both these viruses depend on a viral RNA polymerase to express proteins. Although these viruses differ in viral structure and replication cycle, there is no doubt that they share many similarities in the symptoms, in particular, the damage to lung cells. There is currently a lack of effective antiviral agents, particularly for the clinical treatment of coronaviruses, and there is significantly high patient morbidity and mortality after infection with these respiratory viruses. The COVID-19 and the previous emerging virus outbreaks highlight the urgent need for broad-spectrum antivirals.

The activation of innate immunity is essential for the host cells to inhibit the infection of viruses and other pathogens through the production of interferons (IFNs) and proinflammatory cytokines. Type I IFNs are essential for the classic antiviral response in mammals. After virus infection, host cells quickly produce IFN-α/βs, and more than a hundred interferon-stimulating genes (ISGs) would be further triggered by the activation of the JAK-STAT pathway [18]. Thus, type I IFNs and ISGs are two important components that provide the first line of defense against viral infections. However, a detailed understanding of the molecular mechanisms of SARS-CoV-2, panH1N1, and H7N9 pathogenesis is still elusive, and exploring the interactions between the host and the pathogens will definitely play an important role in the discovery of antiviral agents.

Recently, the discovery and development of noncoding RNA, LncRNAs, CircRNAs, and microRNAs have been viewed as potential sources of genomic medicine after viral infection, based on their gene silencing functions. Recent studies have demonstrated that miRNAs can act as antiviral therapeutic tools in the restriction of herpes simplex virus (HSV), hepatitis C virus (HCV), influenza virus infection, human immunodeficiency virus-1 (HIV-1), and dengue virus (DENV) [19,20,21]. Therefore, exploring the function of lncRNAs, miRNAs, and circRNAs after viruses invade will assist in constructing a competitive endogenous RNA (ceRNA) network, which may contribute to providing novel and effective targets for the exploration and development of innovative therapeutic strategies.

In past decades, there was considerable research in an attempt to discover broad-spectrum antivirals targeting hosts or viruses [22,23,24]. Most broad-spectrum antivirals target signaling pathways in host cells that are utilized by virus replication cycles or host antiviral immunity [25]. In the current study, we used RNA-seq and enrichment analysis of host cells to reveal multiple molecular mechanism networks and functional categories related to host innate immunity, virus pathogenicity, and cellular responses to respiratory viruses including SARS-CoV-2, panH1N1, and H7N9 infection, to provide a scientific basis for exploring drug targets.

## 2. Materials and Methods

### 2.1. Cell Culture and Virus Stocks

The human lung epithelial A549 cell line and human embryonic kidney (HEK) epithelial 293T cells used in this study were purchased from the American Type Culture Collection (ATCC, Manassas, VA, USA). The A549 cells were maintained in F12-K medium, while 293T cells were cultured in DMEM medium, both of them containing 1% penicillin and streptomycin (PS) and 10% fetal bovine serum (FBS). The virus strains used in this study included SARS-CoV-2 (BetaCoV/Shenzhen/SZTH-003/2020 strain virus GISAID access number: EPI_ISL_406594), H7N9 (A/Shanghai/1/2013), and panH1N1 (A/California/07/2009). The A549 cells in this study were used for virus infection. The cells were infected with SARS-CoV-2, panH1N1, and H7N9 at a multiplicity of infection (MOI) of 0.01. After 1 h, the cells were washed twice with sterile PBS buffer, then an F12-K medium containing 2% fetal bovine serum was added. The cells were collected for analysis at 60 h post-infection. The HEK-293T cells in this study were used for the Dual-Luciferase reporter assay. All cells were cultured at 37 °C in a 5% CO_2_ incubator.

### 2.2. RNA Sequencing and Data Analysis

We performed our RNA sequencing and analysis at CapitalBio Technology (Beijing, China). Cells were infected with SARS-CoV-2, panH1N1, and H7N9 at an MOI of 0.01 for 60 h (post infection), respectively. We used TRIzol reagent to extract total RNAs from the control and virus-infected cells, according to the manufacturer’s instructions (Invitrogen, Carlsbad, CA., USA). The genomic DNA in the extracted RNAs was removed using DNase I (Takara, Japan). An RNA 6000 Pico kit (Agilent, Palo Alto, CA, USA) was used to assess the quality of the RNA samples, and RNA integrity was further examined using agarose gel electrophoresis. Ribosomal RNA (rRNA) was removed from the total RNA using Epicentre Ribo-ZeroTM rRNA Removal Kit (Epicentre, Charlotte, NC, USA) following the manufacturer’s instructions. We used the high quality of RNA [RNA integrity number (RIN) ≥ 8] to construct the sequencing library with an Illumina TruSeq Stranded mRNA Library Preparation kit (Illumina, Carlsbad, CA, USA). We using a MiSeq instrument (Illumina) with paired-end libraries (CapitalBio, http://cn.capitalbio.com/, accessed on 13 May 2020) to identified the transcriptional changes after RNA sequencing. We performed three replicates of treatment, and the results were analyzed independently. We used FastQ (Version 0.10.1) to assess the quality of the raw reads. We normalized the expression of each gene using the number of reads equivalent to mapped reads [reads per kilobase per million mapped reads (RPKM)]. We assessed the quality of the obtained data based on the presence and abundance of contaminating sequences, the GC content and the average read length. With the exception of the microarray design, the NGS experiment conformed to the MIAME guidelines [26]. 

The NEBNext Multiplex Small RNA Library Prep Set for Illumina (NEB, Houston, TN, USA) was used for miRNA library preparation; all cDNA libraries were collected from three replications of cells experiments. 1 µg of total RNA input was used to generate cDNA libraries using TruSeq Small RNA Library Prep Kit (Illumina). miRNA DeepSeq was performed using an Illumina HiSeq 2500 instrument at the 50-bp single-end condition. 

CuffDiff analysis was then used to identify the dysregulated RNAs [27]. The RNAs with a fold change ≥2 and a false discovery rate (FDR) of <0.05 were considered to be differentially expressed. Gene ontology (GO) and Kyoto Encyclopedia of Genes and Genomes (KEGG) enrichment analyses were performed for differentially expressed genes. Blast2go and omicsbean (http://www.omicsbean.cn/, accessed on 7 June 2020) were used for GO, and the KEGG Automatic Annotation Server (KAAS) was used for KEGG.

### 2.3. Viral RNA Extraction and qRT-PCR Experiments

Viral RNAs from cell culture supernatant were extracted using the QIAamp RNA Viral Kit (Qiagen, Heiden, Germany). The extracted RNA was then reverse transcribed to cDNA using the PrimeScript II cDNA synthesis Kit (Takara, Japan). Specific primers were used to amplify the cDNA (Supplementary Table S1) and to subsequently detect SARS-CoV-2, panH1N1, and H7N9. Real-time qPCR experiments were performed using a LightCycler^®^ 96 system (Roche, Nutley, NJ, USA). The amplification conditions were incubation at 50 °C for 10 min, Taq activation for 5 min at 95 °C, followed by 38 cycles of amplification comprising denaturation for 10 s at 95 °C, annealing, and primer extension for 40 s at 55 °C. 

### 2.4. Gene Expression Assay by Real-Time qPCR

A549 cells were collected at 60 h post infection. We performed the qRT-PCR assays according to previous procedures [28]. Briefly, Total RNA was isolated with TRIzol reagent (Invitrogen, USA) and was immediately reverse-transcribed using Prime Script™ RT reagent Kit with gDNA Eraser (TaKaRa, Japan, RR047). The mRNA level was measured by qRT-PCR using the TB Green Premix Ex Ta (Takara, Japan, RR42LR) on the CFX96 Touch apparatus (Bio-Rad, Hercules, CA, USA). The results were standardized to the expression of human GAPDH mRNA expression. The specific primers used in this study are listed in Supplementary Table S2. The PCR cycling conditions used are as follows: initial denaturation at 95 °C for 5 min and then denaturation at 94 °C for 30 s, annealing at 60 °C for 45 s, and extension at 72 °C for 30 s. The above procedures were repeated for 35 cycles with a final extension at 72 °C for 5 min. The relative expression was calculated using the 2^−ΔΔCT^ method.

### 2.5. Dual-Luciferase Reporter Assay

We subcloned the complementary DNA sequence into the psiCHECK2 vector (Promega, Madison, WI, USA) to determine the binding relationship and even the binding site among lncRNA, miRNA, and mRNA as previously described [29]. Briefly, a fragment sequence containing the potential binding sites of lncRNA-34087.27 was conducted into a 3′UTR luciferase reporter psiCHECK2 plasmid (C8021, Promega, USA) named h-Lnc34087.27-WT; a mutant sequence of the plasmid of lncRNA- XLOC_098131 without the predicted miR-302b-3p binding sites named h-Lnc34087.27-mut. The 3′-UTR of IRF1 as well as a mutant sequence were also subcloned into the psiCHECK2 plasmid (C8021, Promega, USA) named h-IRF1-WT and h-IRF1-mut. The has-mir-302b-3p plasmids overexpression was conducted in pMIR-REPORT (AM5795, Invitrogen, USA). These plasmids were constructed by Sangon Biotech Company (Shanghai, China) to test the ability of lncRNA to bind to mir-302b-3p. A total of 293T cells were seeded (1 × 10^5^) into 24-well plates for 24 h, and then the luciferase reporter vectors were transfected into 293T cells by using the Lipofectamine 3000 transfection reagent (Invitrogen, Carlsbad, CA, USA). Cells, after transfection, were incubated for 48 h, and the luciferase activities were measured by the Dual-Luciferase Reporter Assay System (Promega, USA). The relative luciferase activity was normalized to Renilla luciferase.

### 2.6. Western Blot Assay

The A549 cells were collected 60 h post of infection. The cells were lysed with radioimmunoprecipitation assay (RIPA) lysis buffer with protease inhibitors (Beyotime, Shanghai, China) added to collect total proteins. The proteins were loaded onto SDS-PAGE gels (10–15%), electrophoresed, and then transferred to polyvinylidene difluoride (PVDF) membranes (CWBiotech, Beijing, China) through an electrophoretic transfer chamber (Millipore, Temecula, CA, USA). The membranes were washed with Tris-buffered saline (TBS) containing 0.1% Tween-20 (TBST) three times and blocked with 5% nonfat milk in TBST at 37 °C for 2 h. Subsequently, the membranes were incubated with primary antibodies at 4 °C overnight and then incubated with horseradish peroxidase (HRP)-conjugated secondary antibodies for 1 h at room temperature (Goat anti-Rabbit IgG: Abcam, ab205718, 1:5000 dilution). The primary antibodies used were: GAPDH: CST, 5174, 1:1000 dilution; IRF1: CST, 8478, 1:1000 dilution. Finally, the immunoblot bands were visualized with an enhanced chemiluminescence (ECL) kit (Beyotime, China) and read using a chemiluminescence system (Thermo Scientific, Waltham, MA, USA).

### 2.7. Statistical Analysis

Univariate analysis of variance (ANOVA) was used for statistical analyses followed by two-tailed Student’s *t*-tests as appropriate. The data are expressed as the mean standard error of the mean. *p* < 0.05 was considered significant, and *p* < 0.01 was considered highly significant. All statistical analyses were performed using SPSS v19.0 and GraphPad Prism 6 software. 

## 3. Results

### 3.1. Comparative Analysis of the Gene Expression Profiles after SARS-CoV-2, panH1N1, and H7N9 Infection

**Figure 1:**
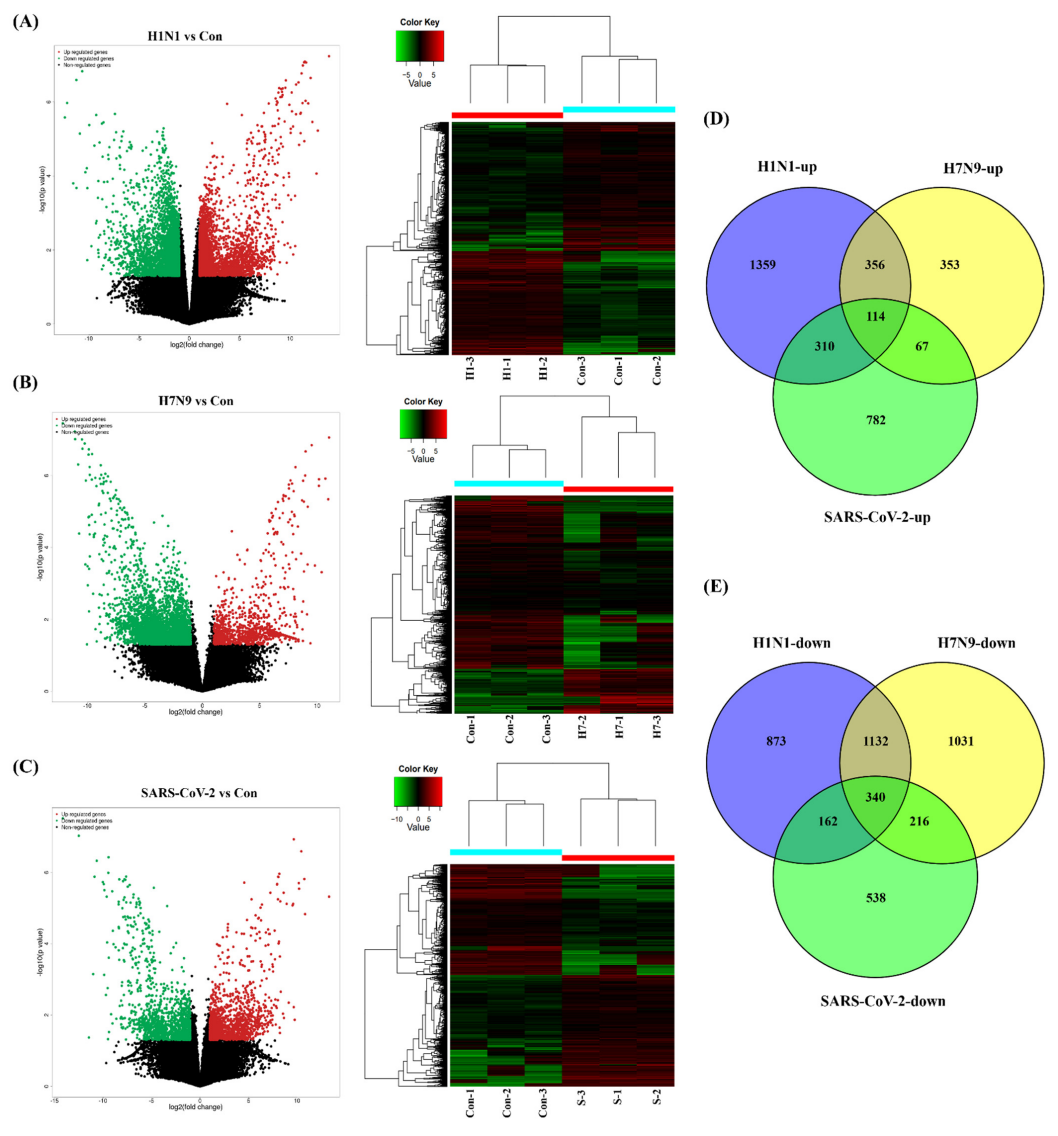
Clustering analysis of viral infections and control based on mRNA RNA-seq. (**A**–**C**) Volcano plots and heatmaps of differentially expressed genes (DEGs) after SARS-CoV-2, panH1N1, and H7N9 invaded. “H1-1-3” represents the H1N1 infection group with 3 replications, “H7-1-3” represents the H7N9 infection group with 3 replications, “S-1-3” represents the SARS-CoV-2 infection group with 3 replications. (**D**,**E**) A Venn analysis of all the upregulated and downregulated mRNAs among the three groups, respectively.

First, one-step growth curves of SARS-CoV-2, panH1N1, and H7N9 in A549 cells were measured. All three viruses were able to infect and replicate in A549 cells and reached a peak within 60 h post incubation (h.p.i.) (Figure S1). Notably, panpanH1N1 and H7N9 replicated to high titers of 5.03 × 10^7^ and 1.94 × 10^7^ copies/μL, respectively, while only 1.06 × 10^3^ copies/μL were produced for SARS-CoV-2 (Figure S1). To compare the profiles of cellular genes after SARS-CoV-2, panH1N1, and H7N9 infection and further explore the possible differences in viral pathogenesis, RNA-seq datasets were obtained utilizing the A549 cell infection model. We established nine cDNA libraries from SARS-CoV-2-, panH1N1-, and H7N9-infected cells collected at 60 h.p.i. with three replicates for each group and identified 149,777 mRNAs in total. Compared to the control group, 2139 mRNAs were upregulated, and 2507 were downregulate in the panH1N1 infected group, while 890 mRNAs were upregulated and 2719 were downregulated in the H7N9 infected group. Additionally, there were 1273 mRNAs that were upregulated, and 1256 were downregulated in the SARS-CoV-2 infected group (Figure 1A–C). Using Venn diagram analysis, a total of 114 upregulated and 340 downregulated mRNAs overlapped among the panH1N1, H7N9, and SARS-CoV-2 infected groups, and 356 upregulated and 1132 downregulated mRNAs overlapped in the panH1N1 and H7N9 infected group, which indicated there were more similarities between the two groups. Moreover, 782 upregulated and 538 downregulated mRNAs were specifically found in SARS-CoV-2 infection (Figure 1D,E). 

### 3.2. Functional Categorization and Pathway Analysis of the Common Differentially Expressed Genes (DEGs) among the Three Viral Infections

**Figure 2:**
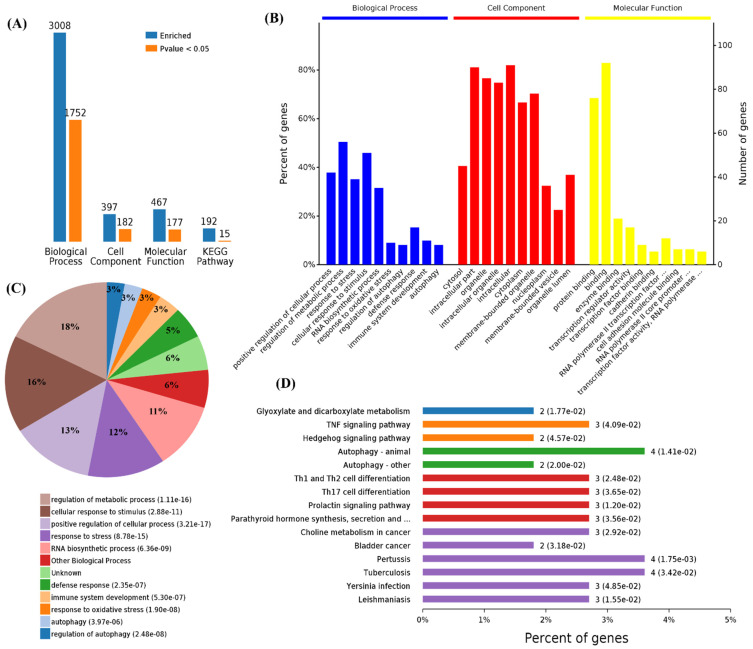
Functional enrichment of the 114 commonly upregulated mRNAs among infected groups. (**A**) Number of commonly differentially expressed genes enriched in GO terms and KEGG pathways. (**B**) Overview of significantly enriched biological process, cell component, and molecular function of the commonly upregulated genes. (**C**) Pie chart of an enriched biological process of the commonly upregulated genes. (**D**) Percentage of commonly upregulated genes associated with enriched KEGG pathways.

First, we performed functional enrichment analyses using 114 commonly upregulated genes. A total of 3008 terms associated with biological process (BPs), 397 terms for cell components (CCs), 467 terms for molecular functions (MFs), and 192 terms for Kyoto Encyclopedia of Genes and Genomes (KEGG) pathways were enriched, and 1752, 182, 177, and 15 of these terms, respectively, showed statistically significant differences (Figure 2A). From the analysis of GO terms, it was shown that the three viruses mainly affected biological processes in the positive regulation of the cellular process, regulation of the metabolic process, response to stress, cellular response to stimulus, RNA biosynthetic process, response to oxidative stress, regulation of autophagy, defense response, immune system development, and autophagy (Figure 2B,C). For the cell components, the three viruses mainly affected the cytosol, intracellular part, organelle, intracellular organelle, intracellular, cytoplasm, membrane-bounded organelle, nucleoplasm, membrane-bounded vesicle, and organelle lumens (Figure 2B). Furthermore, viral infections also affected the molecular functions in protein binding, binding, enzyme binding, transcription regulator activity, transcription factor binding, cadherin binding, and RNA polymerase II transcription factor activity in these cells (Figure 2B). Specifically, 1752 differential BPs were further analyzed. On the basis of the number of enriched genes, the upregulated genes in the viral infection groups were mainly enriched in the regulation of metabolic processes (56 genes), cellular responses to a stimulus (51 genes), positive regulation of cellular processes (42 genes), and RNA biosynthetic processes (35 genes). From the analysis of the KEGG pathway of 114 commonly upregulated genes, it was found that they were mainly involved in autophagy, pertussis, and tuberculosis (Figure 2D). 

**Figure 3:**
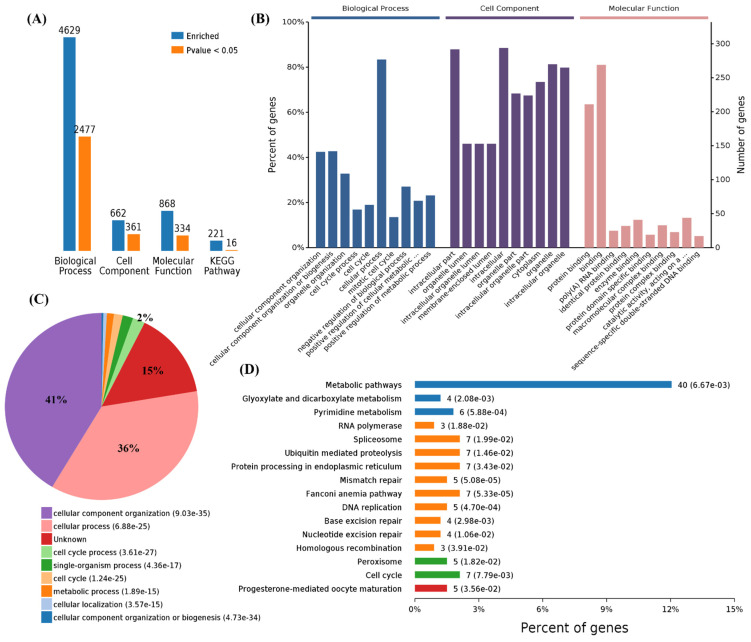
Functional enrichment of 340 commonly downregulated mRNAs among infected groups. (**A**) Number of commonly differentially expressed genes enriched in GO terms and KEGG pathways, blue represents all enriched GO terms, and orange represents significantly enriched GO terms, *p* < 0.05. (**B**) Overview of the significantly enriched biological process, cell component, and molecular function of the commonly downregulated genes. (**C**) Pie chart of an enriched biological process of the commonly downregulated genes. (**D**) Percentage of commonly downregulated genes associated with enriched KEGG pathways.

We also performed a functional enrichment analysis using the 340 downregulated genes, and the results showed 4629, 662, 868, and 221 terms associated with biological process, cell components, molecular function, and KEGG pathways, in which 2477, 361, 334, and 16 terms were significantly enriched, respectively (Figure 3A). The analysis of the GO terms revealed that SARS-CoV-2, panH1N1, and H7N9 infection mainly affected BPs in the cellular component organization, cellular component organization or biogenesis, organelle organization, cell cycle processes, cell cycle, cellular processes, mitotic cell cycle, and negative regulation of biological processes (Figure 3B,C). The viral infection mainly affected CCs in the intracellular part, organelle lumen, intracellular organelle lumen, membrane-enclosed lumen, intracellular organelle part, and intracellular organelle part and cytoplasm (Figure 3B). The virus infection also affected the MFs in protein binding, binding, and poly(A) RNA binding in these cells (Figure 3B). 

A total of 2477 differential BPs were further analyzed. On the basis of the number of enriched genes, the downregulated genes in the viral infection groups were mainly enriched in the cellular process (277 genes), cellular component organization or biogenesis (142 genes), cellular component organization (141 genes), and also cell cycle (63 genes) and cell cycle processes (56 genes). A pie chart of the BP distribution is shown in Figure 3D. From the analysis of the KEGG pathway of 340 overlapping downregulated genes, it was found that they were mainly involved in metabolic pathways, such as the cell cycle and spliceosome, and among others (Figure 3D). 

### 3.3. Functional Categorization and Pathway Analysis of Specific DEGs in SARS-CoV-2 Infection

**Figure 4:**
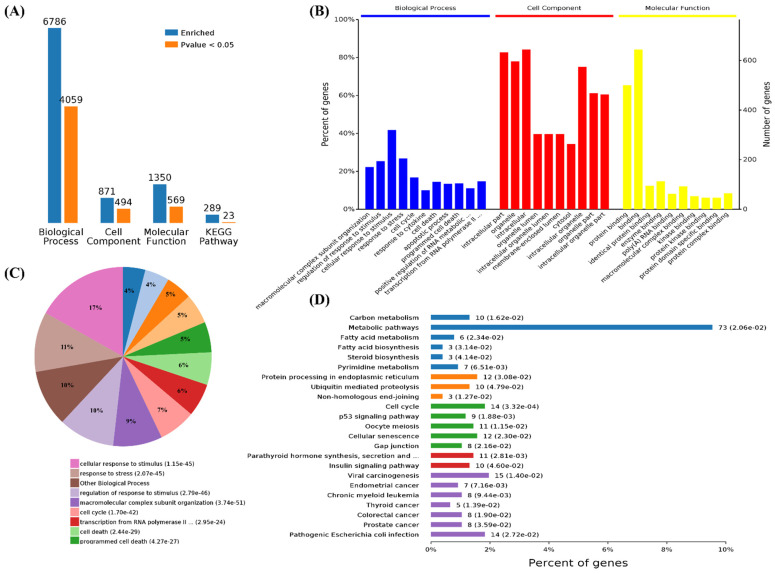
Functional enrichment and KEGG pathway analysis of SARS-CoV-2 alone induced upregulated genes. (**A**) Number of upregulated genes enriched in GO terms and KEGG pathways, blue represents all enriched GO terms, and orange represents significantly enriched GO terms, *p* < 0.05. (**B**) Overview of significantly enriched biological process (in blue), cell component (in red), and molecular function (in yellow) of the upregulated genes induced by SARS-CoV-2 infection. (**C**) Pie chart of an enriched biological process of the upregulated genes. (**D**) Percentage of genes related to enriched KEGG pathways.

**Figure 5:**
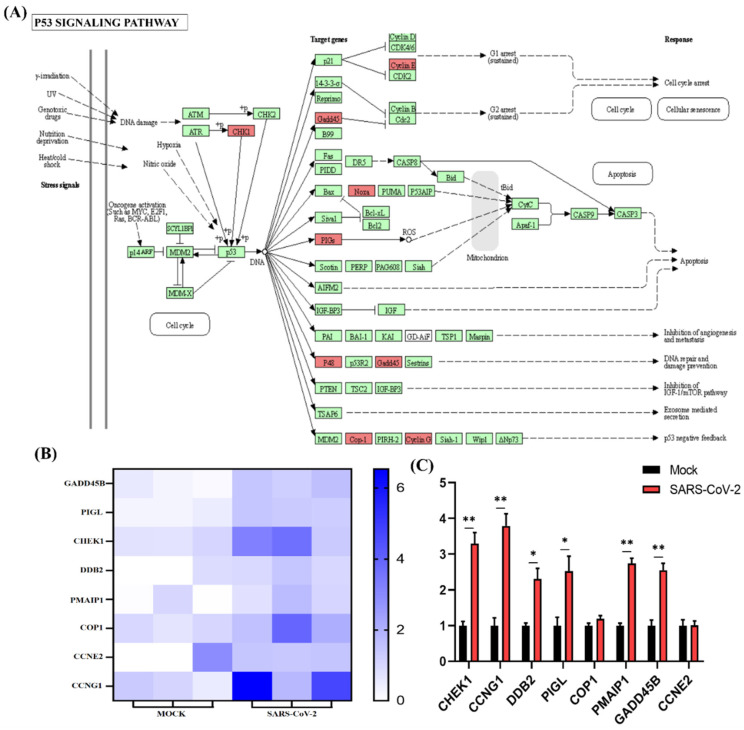
P53 signaling pathway analysis of SARS-CoV-2 alone induced upregulated genes. (**A**) SARS-CoV-2 infection could regulate the P53 signaling pathway. The key upregulated genes induced by SARS-CoV-2 infection are marked in red. (**B**) Expression pattern of upregulated genes induced by SARS-CoV-2 infection involved in P53 signaling pathways. (**C**) Relative RNA expression quantified by qRT-PCR, *n* = 3. “*” represents *p* < 0.05 and “**” represents *p* < 0.01.

**Figure 6:**
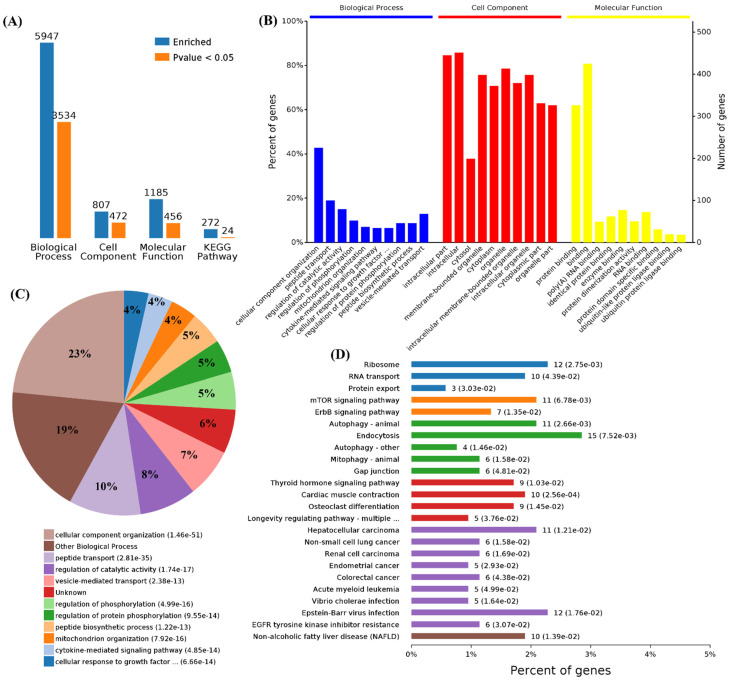
Functional enrichment and KEGG pathway analysis of SARS-CoV-2 alone induced downregulated genes. (**A**) Number of downregulated genes enriched in GO terms and KEGG pathways. (**B**) Overview of significantly enriched biological process, cell component, and molecular function of the downregulated genes induced by SARS-CoV-2 infection. (**C**) Pie chart of enriched biological processes of the downregulated genes. (**D**) Percentage of genes related to enriched KEGG pathways.

**Figure 7:**
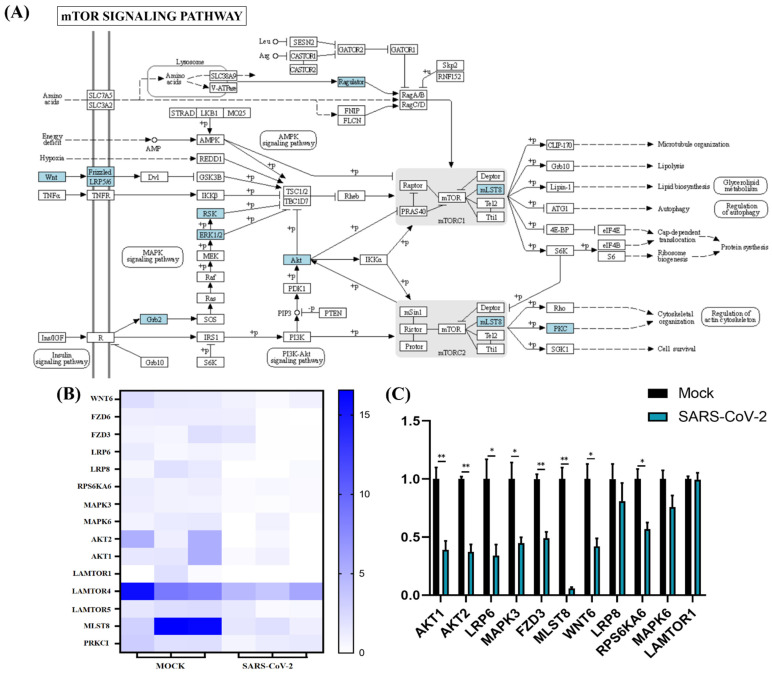
mTOR signaling pathway analysis of SARS-CoV-2 alone induced downregulated genes. (**A**) SARS-CoV-2 infection could regulate the mTOR pathway. The key downregulated genes are marked in blue. (**B**) Expression pattern of downregulated genes involved in mTOR signaling pathways. (**C**) Relative RNA expression quantified by qRT-PCR, *n* = 3. “*” represents *p* < 0.05 and “**” represents *p* < 0.01.

Functional enrichment analysis was further performed using the 782 upregulated genes and the 538 downregulated genes specifically found in SARS-CoV-2 infection. For the upregulated genes, 4059, 494, 569, and 23 terms in BPs, CCs, MFs, and KEGG were significantly enriched, respectively (Figure 4A). For the downregulated genes, 1534, 472, 456, and 24 terms in BPs, CCs, MFs, and KEGG were significantly enriched, respectively (Figure 5A). Of note, GO analysis showed that SARS-CoV-2 infection could upregulate more than 100 genes involved in the cell death, apoptotic process, and programmed cell death terms (Figure 4B,C). Furthermore, KEGG pathway analysis also showed that the upregulated genes were mainly involved in metabolic pathways, viral carcinogenesis, and the cell cycle (Figure 4D). Further analysis showed that nine upregulated genes (upstream) were involved in the p53 signaling pathway (Figure 5A) and were verified by qRT-PCR analysis (Figure 5B,C), indicating that host cell death and the cell cycle play important roles in SARS-CoV-2 infection. For the specific downregulated DEGs, GO term analysis revealed that they were mainly enriched in the cellular component organization, peptide transport, regulation of catalytic activity, regulation of phosphorylation, mitochondrion organization, cytokine-mediated signaling pathway, cellular response to growth factor, regulation of protein phosphorylation, peptide biosynthetic process, and vesicle-mediated transport (Figure 6C). The KEGG pathway analysis showed that the downregulated genes were mainly involved in endocytosis, ribosome, the mTOR signaling pathway, and autophagy (Figure 6D). Similarly, further analysis showed that at least 15 downregulated genes were involved in the mTOR signaling pathway, which was verified by qRT-PCR analysis (Figure 7). These data indicated that host cell death and autophagy might play important roles in SARS-CoV-2 infection.

**Figure 8:**
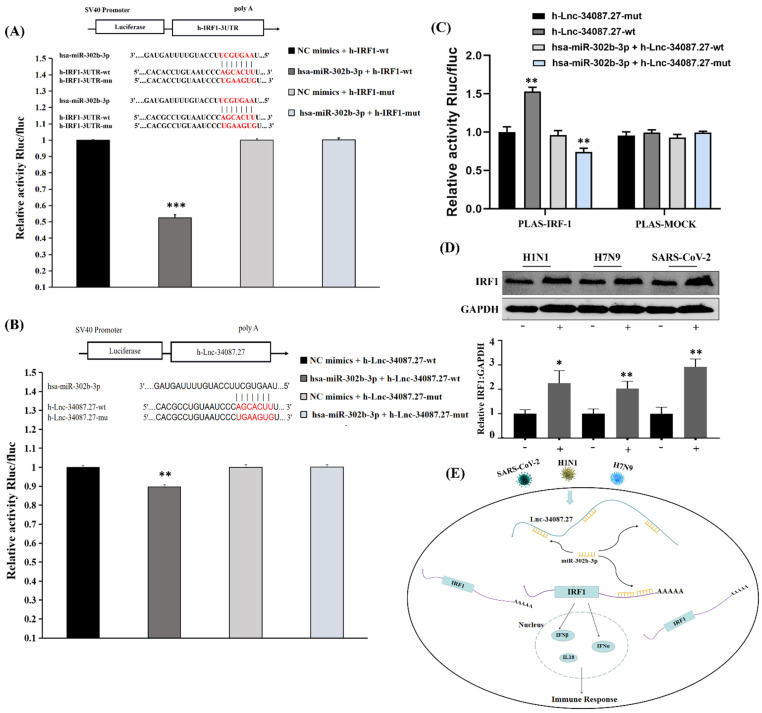
LncRNA-34087.27 upregulates IRF1 expression by acting as a competitive endogenous RNA. (**A**) Relative activity of Rluc/fluc in HEK293T cells cotransfected with miR-302b-3p and IRF1 3′-UTR. “***” represents *p* < 0.001. (**B**) Relative activity of Rluc/fluc in HEK293T cells cotransfected with miR-302b-3p and lncRNA-34087.27. “**” represents *p* < 0.01. (**C**) Relative activity of Rluc/fluc in HEK293T cells cotransfected with IRF1 and lncRNA-34087.27, lncRNA-34087.27-mut, lncRNA-34087.27 + miR-302b-3p, or lncRNA-34087.27-mut + miR-302b-3p. “**” represents *p* < 0.001. (**D**) The protein expression of IRF1 after SARS-CoV-2, panH1N1, and H7N9 infection. “*” represents *p* < 0.05 and “**” represents *p* < 0.01. (**E**) Summary of the potential mechanisms of a competitive endogenous RNA network that affects the immune responses in SARS-CoV-2-, panH1N1-, and H7N9-infected cells.

To highlight the changes in the cellular host genes after SARS-CoV-2 invaded and to further explore the molecular mechanism of viral pathogenesis, we identified several related studies through electronic databases (GEO databases or PubMed). It is worth mentioning that we found that IRF1 was significantly upregulated in SARS-CoV-2-infected lung cells [30,31,32,33,34], which indicated that IRF1 might play an important role in inflammatory and interferon response after SARS-CoV-2 infection (Figure S2). Moreover, the protein level of IRF1 was significantly increased by SARS-CoV-2, panH1N1, and H7N9 infection (Figure 8D).

### 3.4. LncRNA-34087.27 Upregulates IRF1 Expression by Acting as a Competitive Endogenous RNA in SARS-CoV-2, panH1N1, and H7N9 Infections

To gain deeper insights into the transcriptome of the three viral infections, we further analyzed the expression profiles of lncRNAs, circRNAs, and microRNAs. We identified 1010, 4696, and 6925 significantly upregulated lncRNAs and 1094, 3920, and 2082 significantly downregulated lncRNAs in SARS-CoV-2-, panH1N1-, and H7N9-infected cells, respectively. The results of the Venn diagram analysis showed that 83, 7, and 2 commonly upregulated and 88, 22, and 4 commonly downregulated lncRNAs, circRNAs, and microRNAs were found, respectively (Figures S3 and S4 and Tables S3–S5). 

Next, we analyzed the potential binding sites of these differentially expressed lncRNAs, microRNAs, and mRNAs using mirDB (http://www.mirdb.org/miRDB/, accessed on 3 February 2020) Notably, lncRNA-34087.27 has four binding sites for miR-302b-3p (Figure S5, Table S6), and it also had four binding sites for IRF-1, suggesting that lncRNA-34087.27 could regulate the expression of IRF1 by competitively adsorbing miR-302b-3p. To assess the direct binding relationship and even the binding site between miR-302b-3p, IRF1, and lncRNA-34087.27, we constructed luciferase reporters the IRF1-3′-untranslated region (UTR) and the lncRNA-34087.27 that contained mutated miR-302b-3p binding sites or wild-type (WT) sites. We found that upregulation of miR-302b-3p significantly reduced the luciferase activities of the WT reporter vector but had no influence on the empty vector and the mutant reporter vector (Figure 8A,B). Then, we further explored whether lncRNA-34087.27 modulated IRF1 through miR-302b-3p. As shown in Figure 8C, overexpression of lncRNA-34087.27-WT increased the transcript level of IRF1, but not the mutant type. In addition, the co-transfection of lncRNA-34087.27-mutant with miR-302b-3p significantly decreased the transcript level of IRF1. Sufficient miR-302b-3p and lncRNA-34087.27-WT expression had no impact on the luciferase activity of the IRF1 luciferase vector compared with that of the control, while the above treatments had no influence on the luciferase activity in the mock. A summary of the potential mechanisms of the competitive endogenous RNA network on the immune responses in SARS-CoV-2-, panH1N1-, and H7N9-infected cells is shown in Figure 8E.

## 4. Discussion

The outbreak of the novel coronavirus, SARS-CoV-2, responsible for the current COVID-19 pandemic, has caused a worldwide public health emergency. The receptor-binding domain (RBD) of SARS-CoV-2 spike (S) protein mediates receptor binding and fusion of the viral and cellular membrane via angiotensin-converting enzyme 2 (ACE2) [17]. There were so many variants of SARS-CoV-2 encoding mutations in the RBD of spike protein that have been identified, including B.1.1.7 (Alpha) [35], B.1.351 (Beta) [36], B.1.617.2 (Delta) [37], P.1 (Gamma) [38], and B.1.1.529 (Omicron) [39], which have been proved with the more efficient transmission. The effectiveness of vaccines and neutralizing antibody drugs has been observed to decline as mutants of SARS-CoV-2 continue to appear [40]. Therefore, it is of great significance to develop drugs based on the infection mechanism of viruses. SARS-CoV-2 infection and pathogenesis are subjects of great interest, but SARS-CoV-2 infection-mediated alteration of post-transcriptional gene regulation is still largely unknown. Moreover, both SARS-CoV-2 and flu viruses could primarily attack the respiratory system and cause similar symptoms. These respiratory RNA viruses all have the risk for an outbreak worldwide, and both are easily mutated. It is important to elucidate the similarities in the pathogenic mechanism between the novel coronavirus and flu viruses to explore broad-spectrum therapeutic drugs. The current study shows that IRF1 was upregulated by SARS-CoV-2, panH1N1, and H7N9 infection, in a miR-302b-3p-dependent manner.

Airway epithelial cells are targets for viral infection and replication, and they also provide the first line of inhibition against virus entry. The interaction between host epithelial cells and invaded viruses often affects the outcome of viral infection, either directly or by regulating the subsequent adaptive immune response. Type I IFNs and ISGs are two important components that provide the first line of defense against viral infections in mammals [41,42,43,44,45]. Interferon regulatory factor-1 (IRF1) is a member of the IFN regulatory factor (IRF) family, which is involved in various physiological and pathological events, including tumor immune surveillance, viral infection, immune system development, proinflammatory injury, and autoimmune diseases [46]. IRF1 controls the induction of type III IFN by many pathogens. IRF1 was discovered as a transcription factor that regulates the expression of antiviral genes by binding to the interferon-stimulated response element (ISRE) in their promoters. Moreover, one recent study showed that IRF1 binds to the promoter region of STAT1 to induce the transcription of ISGs, thus inhibiting hepatitis E virus (HEV) replication [47]. Studies have shown that overexpression of IRF1 can protect some susceptible cells against RNA viruses [48,49]. There were also some studies that showed that IRF1 knockout mice are more susceptible to viral infections [50,51,52]. We found IRF1 mRNA was significantly upregulated in lung cells infected by SARS-CoV-2 (Figure S2), including Calu-3 cells [30], ACE2-A549 cells [31], lung organoid model induced by human pluripotent stem cells [32], and primary human bronchial epithelial cells [33], and then proved by Western-blot, indicating the important role of IRF1 in host immune response after SARS-CoV-2 infection. Moreover, recent studies revealed that inhibiting IRF1 could facilitate H1N1 influenza A virus infection [53], while, in contrast, overexpression of IRF1 suppresses AIV and NDV viral yield in chicken cells [54]. In the current study, we found that IRF1 was significantly upregulated in cells infected by H1N1, H7N9, and SARS-CoV-2, respectively (Figure 8D), and then demonstrated its regulatory mechanisms after viral infection (Figure 8E); a novel lncRNA-34087.27 was upregulated after these respiratory viruses’ infection activated the expression of IRF1 mRNA as a competitive endogenous RNA by adsorbing miR-302b-3p. IRF1 may play an important role in the host to defend against the viral infection, which needs to be further explored.

Autophagy plays different roles in the infection process of various pathogens: autophagy can be activated to combat viruses. However, some pathogens have evolved different strategies to block or even hijack autophagy to promote their replication. Previous studies revealed that autophagy can be induced by the influenza A virus, panH1N1, or H7N9 to facilitate its replication [55,56,57,58]. Moreover, inhibition of autophagy could prevent the replication of SARS-CoV-2 and ameliorate pneumonia in animal models infected by SARS-CoV-2 [59]. It is noteworthy that SARS-CoV-2 may induce autophagy by independently inhibiting the mTOR pathway through the downregulation of *AKT1/2*, *LRP6/8*, *MAPK3/6*, *FZD3*, *MLST8*, *WNT6*, *RPS6KA6*, and *LAMTOR1* mRNA, compared with H1N1 and H7N9 infection, based on our data (Figure 5). A recent study has also shown that SARS-CoV-2 infection inhibits the Akt-mTOR pathway, which is thought to be involved in the induction of autophagy inVero E6 cells [60]; however, ORF3 of SARS-CoV-2 was proven to block the fusion of autophagosomes with lysosomes and to induce incomplete autophagy [55,61]. Indeed, controlling the autophagic process induced by these viruses plays an important role in antiviral activity [62,63,64,65]. However, in the current study, SARS-CoV-2, panH1N1, and H7N9 infection upregulated the expression of autophagy-related genes, such as *ATG7*, *MAPK3*, *MLST8*, *BMF*, and *UCHL1* in A549 cells (https://doi.org/10.5281/zenodo.5489734, accessed on 15 November 2021), which indicated that autophagy is essential for virus pathogenicity, and inhibiting autophagic processes may become a universal antiviral strategy for influenza viruses and SARS-CoV-2 infection. Due to the lack of verification, hypotheses about these targets related to autophagy after SARS-CoV-2 infection requires further exploration.

In summary, the current study compared the virus pathogenic mechanism revealed by host transcriptome profiling after SARS-CoV-2, panH1N1, and H7N9, infection. We demonstrated that SARS-CoV-2, panH1N1, and H7N9 infection could be upregulated by the expression of lncRNA-34087.27, which competitively adsorbed miRNA-302b-3p, ultimately promoting the expression of IRF1 and activating the host’s antiviral immunity. Moreover, we further confirmed that these viruses may induce cell autophagy and that SARS-CoV-2 may independently induce autophagy by inhibiting the mTOR pathway. This study provides new insight into the molecular mechanisms of influenza A and SARS-CoV-2 infection. 

## Data Availability

The RNA sequencing data are openly available in Zenodo (https://doi.org/10.5281/zenodo.5489734). All other data are available upon reasonable request.

## Supplementary Materials

The following supporting information can be downloaded at: https://www.mdpi.com/article/10.3390/cells11030487/s1, Figure S1: Growth curve of SARS-CoV-2, panpanH1N1 and H7N9 after infection; Figure S2: Venn analysis of all up expressed mRNAs among the five SARS-CoV-2 infection groups; Figure S3: Clustering of Viral infection and control based on LncRNA-seq analysis; Figure S4: Clustering of Viral infection and control based on circRNA and microRNAanalysis; Figure S5: Predicted binding sites for IRF-1 3’-UTR and LncRNA-34087.27 with miR-302b-3p; Table S1: Primers for H1N1, H7N9 and SARS-CoV-2 detection; Table S2: Primers for qRT-PCR; Table S3: Top 20 differential expression circRNA of differential infection groups; Table S4: Top 20 differential expression miRNA of differential infection groups; Table S5: Top 20 differential expression lncRNA of differential infection groups. Table S6: The sequence of LncRNA34087.27, IRF1-3’UTR and miR-302b-3p.
