# Supplementary material for: Competitive Endogenous RNA Network Activates Host Immune Response in SARS-CoV-2-, panH1N1 (A/California/07/2009)-, and H7N9 (A/Shanghai/1/2013)-Infected Cells
Source: Cells. 2022 Jan 30;11(3):487. doi: 10.3390/cells11030487 (PMC8834034; doi:10.3390/cells11030487)
Supplement: Supplementary file 1 [file cells-11-00487-s001.zip › cells-1489030-supplementary/Supplemental Materials/Supplemental Figures-Merged.pdf]

# Supplemental Figures

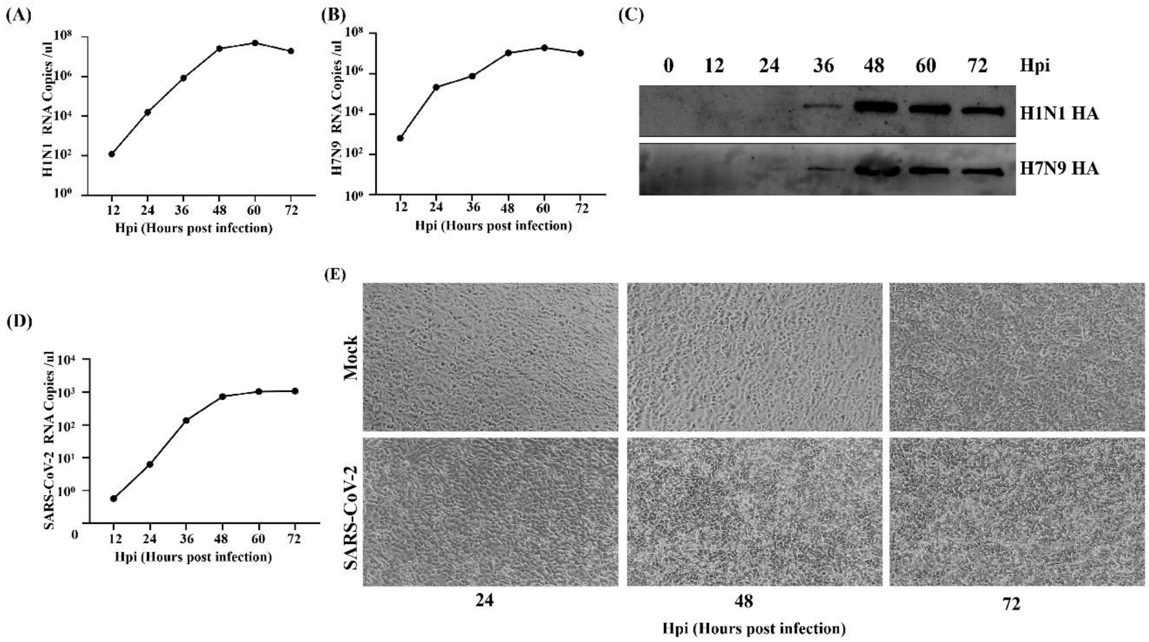


**Figure S1. Growth curve of SARS-CoV-2, panpanH1N1 and H7N9 after infection.** (A-B) The A549 cells were infected with panH1N1 and H7N9 at an MOI of 0.01. Supernatants were harvested at the indicated time points post infection. Viral RNA copies in the supernatants were determined using qRT-PCR, (N=3). Hpi: hours post infection. (C) Viral proteins detection in infected cell lines after infection. HA: Hemagglutinin protein. (D) A549 cell line susceptibility to SARS-CoV-2. The A549 was infected with SARS-CoV-2 at an MOI of 0.01. Supernatants were harvested at the indicated time points post infection. Viral RNA copies in the supernatants were determined using qRT-PCR. (N=3). (E) Cytopathic effects in A549 cells infected with SARS-CoV-2 at indicated time points.

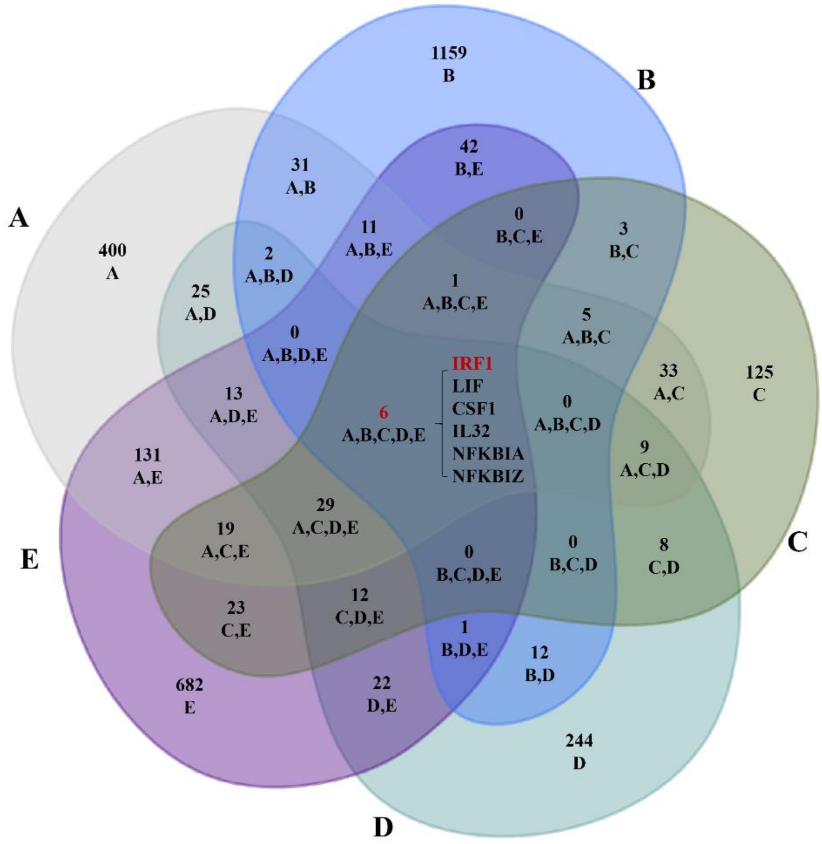


**Figure S2. Venn analysis of all up expressed mRNAs among the five SARS-CoV-2 infection groups.** A: Calu-3 infected with SARS-CoV-2 (doi:10.3390/biology9090260), B: ACE2-A549 infected with SARS-CoV-2 (GSE154613), C: Lung organoid model using human pluripotent stem cells infected with SARS-CoV-2 (GSE155241), D: Primary human bronchial epithelial cells infected with SARS-CoV-2 (GSE150819), E: A549 infected with SARS-CoV-2 (Present study, doi.org/10.5281/zenodo.5489734).

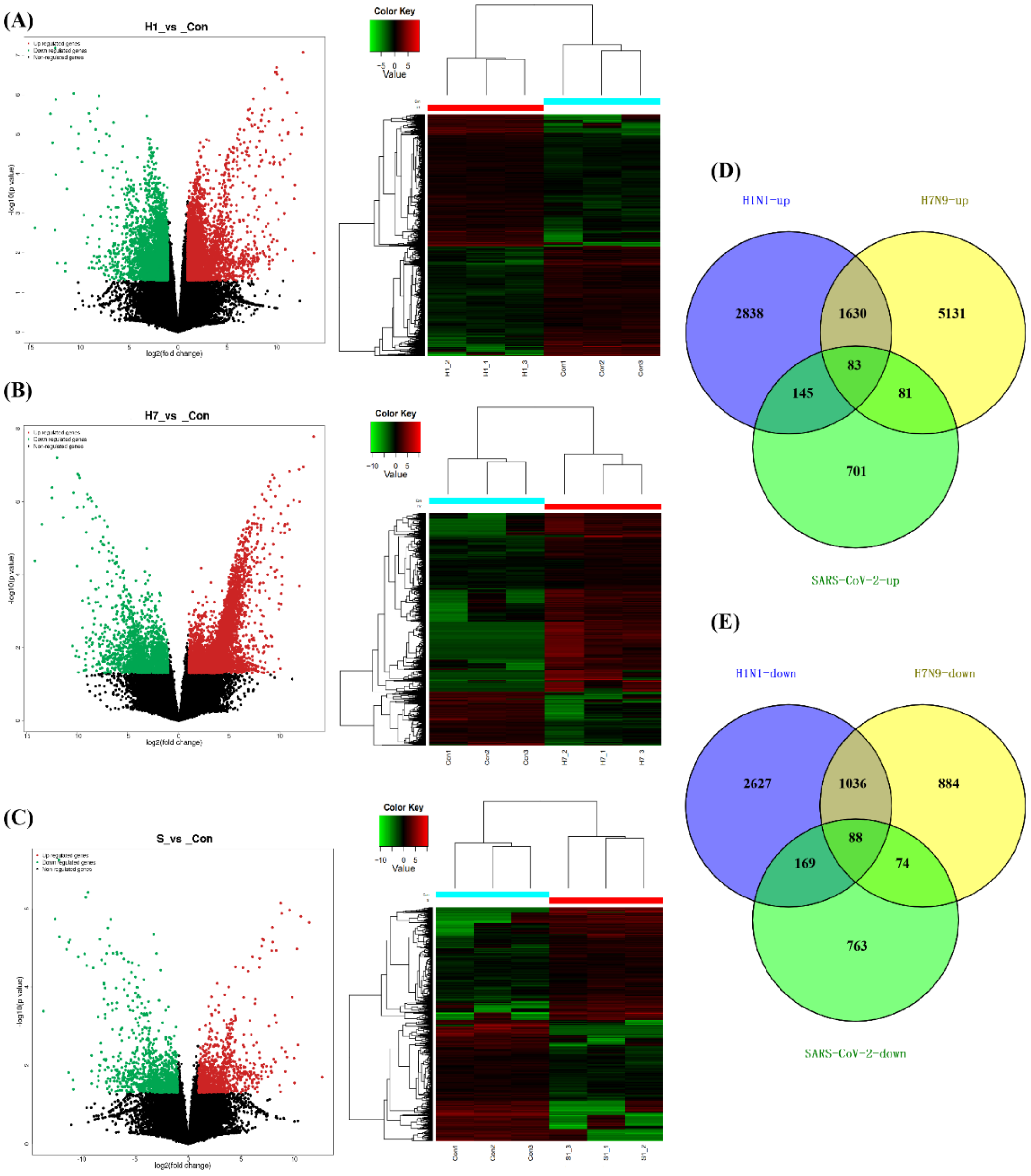


**Figure S3. Clustering of Viral infection and control based on LncRNA-seq analysis.** (A) Volcano and Heatmap of differentially expressed genes (DEGs) in panH1N1 infection group. (B) Volcano and Heatmap of differentially expressed genes (DEGs) in H7N9 infection group. (C) Volcano and Heatmap of differentially expressed genes (DEGs) in SARS-CoV-2 infection group. (D) Venn analysis of all up expressed genes among the three groups. (E) Venn analysis of all down expressed genes among the three groups.

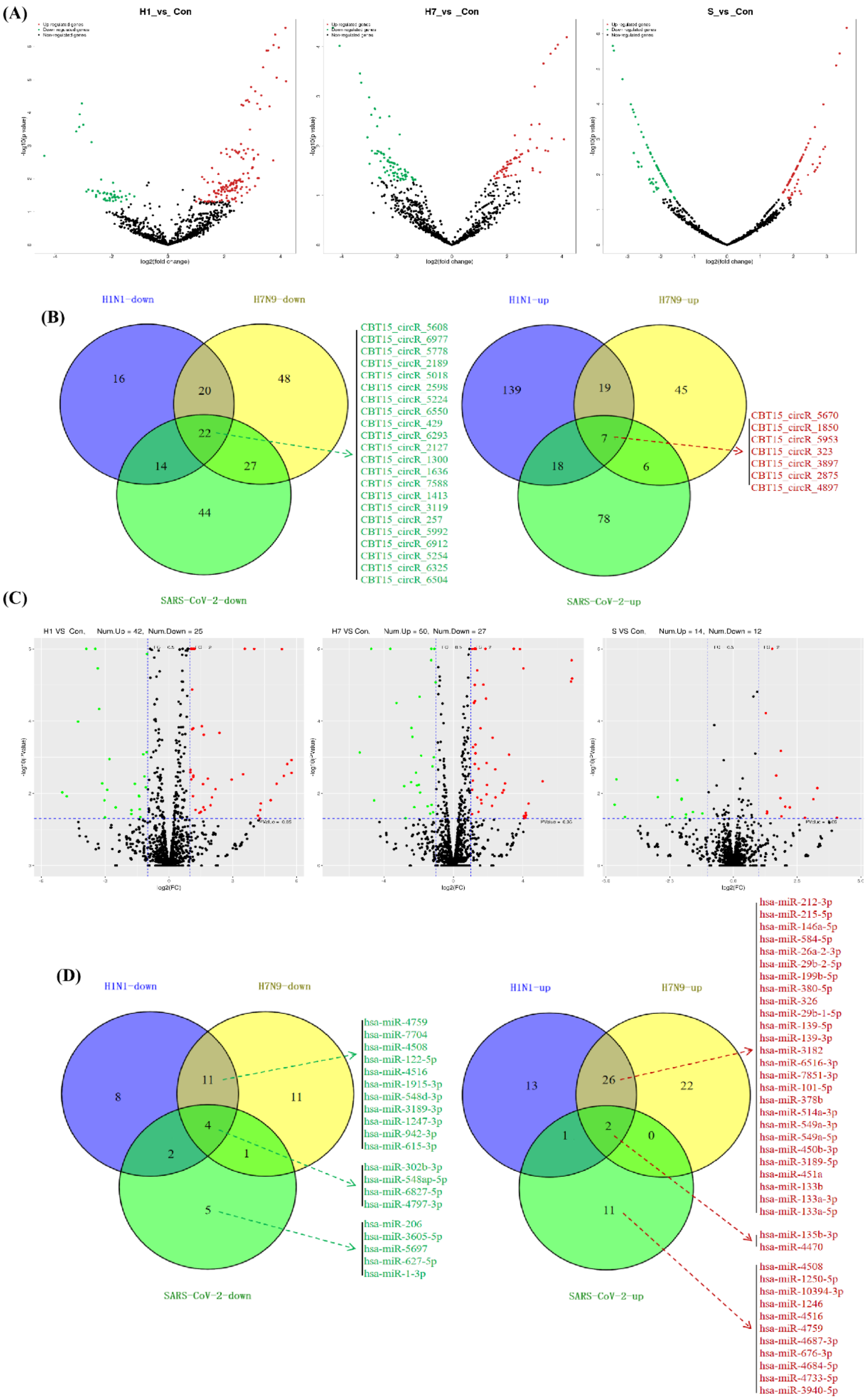


**Figure S4. Clustering of Viral infection and control based on circRNA and microRNA analysis.**

(A) Volcano map of differentially expressed circRNA in panH1N1, H7N9 and SARS-CoV-2

infection groups. (B) Venn analysis of all down/up expressed circRNA among the three groups. (C) Volcano map of differentially expressed miRNA in panH1N1, H7N9 and SARS-CoV-2 infection groups. (D) Venn analysis of all down/up expressed miRNA among the three groups.

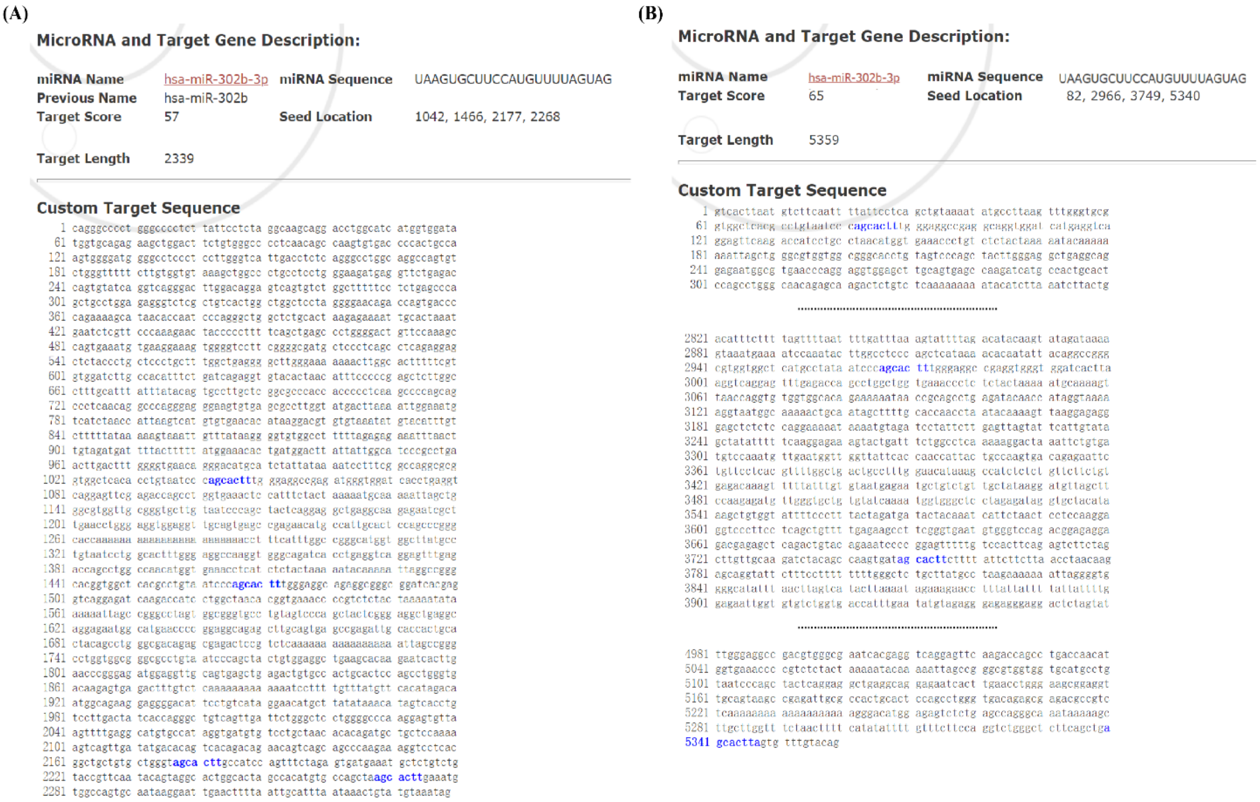


**Figure S5. Predicted binding sites for IRF-1 3'-UTR and LncRNA-34087.27 with miR-302b-3p.** (A) Predicted binding sites of IRF-1 3'-UTR and miR-302b-3p. (B) Predicted binding sites of LncRNA-34087.27 and miR-302b-3p.
