# Supplementary material for: Competitive Endogenous RNA Network Activates Host Immune Response in SARS-CoV-2-, panH1N1 (A/California/07/2009)-, and H7N9 (A/Shanghai/1/2013)-Infected Cells
Source: Cells. 2022 Jan 30;11(3):487. doi: 10.3390/cells11030487 (PMC8834034; doi:10.3390/cells11030487)
Supplement: Supplementary file 1 [file cells-11-00487-s001.zip › cells-1489030-supplementary/Supplemental Materials/Supplemental Tables-Merged.pdf]

Table S1. Primers for H1N1, H7N9 and SARS-CoV-2 detection

Position	Primer Sequence(5'–3')
H1N1-N	Forward: GCACGGTCAGCACTTATYCTRAG
	Reverse: GTGRGCTGGGTTTTCATTTGGTC
	Probe: CYACTGCAAGCCCATACACACAAGCAGGCA
H7N9-HA	Forward: AGAAATGAAATGGCTCCTGTCAA
	Reverse: GGTTTTTTCTTGTATTTTTATATGACTTAG
	Probe: AGATAATGCTGCATTCCCGCAGATG
SARS-CoV-2-S	Forward: TGCAGGTATATGCGCTAGTTATCAG
	Reverse: CACCAAGTGACATAGTGTAGGCAAT
	Probe: TCAGACTAATTCTCCTCGGCGGGCA

Y and R in red are degenerate bases, Y represents T/C, R represents A/G. The primers H1N1-N could be complementary to the complete CDS regions of Influenza A virus (A/California/07/2009(H1N1)) segment 5 nucleocapsid protein (NP) gene. The length of the PCR product was 195 bp. The primers H7N9-HA could be complementary to the complete CDS regions of Influenza A virus (A/Shanghai/02/2013(H7N9)) segment 4 hemagglutinin (HA) gene, The length of the PCR product was 83 bp. The primers SARS-CoV-2-S could be complementary to the complete CDS regions of SARS-CoV-2 SARS-CoV-2 spike glycoprotein gene, the length of the product was 74 bp.

Table S2. Primers for qRT-PCR

Position	Primer Sequence(5'–3')	Tm(°C)	Product size (bp)
CHEK1	Forward: CCAGCCTTCCTTCTTGGGTAT<br>Reverse: AGGTCTTTACGGATGTCAACG	60	415
CCNG1	Forward: CGGCACTTCCTAACCCTGTGT<br>Reverse: TCCACAGGAGGACAGAGCCC	60	722
DDB2	Forward: GGAGGGAACAACTAGGCTGC<br>Reverse: TGTGACCACCATTCGGCTAC	60	124
PIGL	Forward: GATGCAGGGGGAGTAAGTGG<br>Reverse: GCACAGAGCACCCTTTAGGT	60	100
COP1	Forward: AGCAGCTCCAGCCTAGGCA<br>Reverse: TCAAAGCAGATGGGGCATACG	60	119
PMAIP1	Forward: GGGCTCAAGCACAGACTGCA<br>Reverse: GAAGGAGTCCCCTCATGCAAG	60	181
GADD45B	Forward: AACAAAGTCGGGCTGACTGG<br>Reverse: CCCTGTGTGGGGAAAACAGT	60	130
CCNE2	Forward: GACTGCTGCTGCCTTGTGC<br>Reverse: GTCTTCAGCTTCACTGGACTAG	60	147
AKT1	Forward: TCATCGAACGCACCTTCCATG<br>Reverse: CCAGGGACACCTCCATCTCTT	60	168
AKT2	Forward: ATGTACGAGATGATGTGCGGC<br>Reverse: ACCTGTGCTCCATGACCTCC	60	205
LRP6	Forward: GTATCCCTGTGGCTTGGCGG<br>Reverse: GCCCACTGGCACACTGGAAC	60	108
MAPK3	Forward: GTCCACCCTGGAAGCCATGA<br>Reverse: CTTAAGGTCGCAGGTGGTGTT	60	217
FZD3	Forward: CCCCTGATGATCTGTCGGCATC<br>Reverse: TTTGGCATCAGGTGCAGAACAA	60	108
MLST8	Forward: CCGCCCCAGAACTTTGGTCT<br>Reverse: CGAGGCCGAGCGTAGAGTTAT	60	91
WNT6	Forward: AAGGCCTTTGGACGCATCC		

	Reverse: AGAACAGGCCTGCGTGACG	60	102
LRP8	Forward: CTGCCGCTGTTATCGGGATC<br>Reverse: GCAGTTCTCCCTATATGGAGCTC	60	181
RPS6KA6	Forward: GGCCCTAAGGCCCACAAGAA<br>Reverse: CATGAAGGGGCTGGAAAGCCA	60	100
MAPK6	Forward: ATTGACATGTGGGCTGCAGG<br>Reverse: TACGCTGAGAAGCTCCTGACG	60	150
LAMTOR1	Forward: TGCAGACTCACAGGGCATGG<br>Reverse: TCACTGGCCAGCACTTGGTG	60	156
GAPDH	Forward: GATTCCACCCATGGCAAATTCC<br>Reverse: TCGCTCCTGGAAGATGGTGAT	60	93

Table S3-1. Top 20 differential expression circRNA of panH1N1 infection

Running_number	LogFC	Pvalue	Locus	Best transcript
CBT15_circR_2842	-4.39	0.002013761	85633913-85634132	ENST00000253458
CBT15_circR_6977	-3.25	0.00036949	18799294-18804898	ENST00000286485
CBT15_circR_5608	-3.14	0.000270672	74174505-74201370	ENST00000325278
CBT15_circR_3479	-3.13	0.000112002	36026899-36033837	ENST00000357384
CBT15_circR_4645	-3.05	5.25983E-05	62905886-62914406	ENST00000266070
CBT15_circR_4035	-3.00	0.00023083	177231888-177234271	ENST00000397062
CBT15_circR_1722	-2.89	0.024447854	69800208-69801721	ENST00000247833
CBT15_circR_6167	-2.82	0.02140074	149369908-149379518	ENST00000367456
CBT15_circR_3154	-2.71	0.000778249	47664158-47668410	ENST00000290158
CBT15_circR_5964	-2.68	0.023505514	43292473-43297166	ENST00000325110
CBT15_circR_2011	4.19	1.11128E-05	32090501-32117287	ENST00000345122
CBT15_circR_5670	4.17	2.70177E-07	1057470-1065478	ENST00000264930
CBT15_circR_1279	3.93	1.06566E-06	68386315-68390052	ENST00000294304
CBT15_circR_3557	3.86	8.65275E-06	79448781-79451816	ENST00000253506
CBT15_circR_679	3.81	4.2813E-07	110506934-110510596	ENST00000369583
CBT15_circR_1850	3.75	8.98507E-07	32517856-32527532	ENST00000357505
CBT15_circR_763	3.74	0.002787258	132404625-132406289	ENST00000305233
CBT15_circR_323	3.60	6.47053E-05	2302977-2304585	ENST00000378536
CBT15_circR_5953	3.55	1.30912E-06	38990948-39003622	ENST00000296782
CBT15_circR_1273	3.50	1.30815E-06	68347846-68348243	ENST00000294304

Table S3-2. Top 20 differential expression circRNA of H7N9 infection

Running_number	LogFC	Pvalue	Locus	Best transcript
CBT15_circR_7564	-4.33	4.17105E-05	13845-13998	ENST00000361567
CBT15_circR_994	-4.08	9.65834E-05	89751345-89762835	ENST00000260753
CBT15_circR_4207	-3.34	0.000347821	241632916-241634079	ENST00000407315
CBT15_circR_4865	-3.30	0.000532228	45689711-45702847	ENST00000252934
CBT15_circR_6977	-3.07	0.00674829	18799294-18804898	ENST00000286485
CBT15_circR_5608	-3.01	0.001049121	74174505-74201370	ENST00000325278
CBT15_circR_6771	-3.00	0.023084336	80789305-80810701	ENST00000265361
CBT15_circR_7239	-2.93	0.002365954	116331243-116335074	ENST00000328252
CBT15_circR_1300	-2.91	0.012684471	70354300-70356254	ENST00000253925
CBT15_circR_5812	-2.81	0.001773453	143054438-143057747	ENST00000274498
CBT15_circR_3388	4.17	6.42592E-05	81913773-81915681	ENST00000328666
CBT15_circR_5820	4.08	0.007386321	143399655-143400852	ENST00000231509
CBT15_circR_1942	3.85	9.15704E-05	102040235-102040673	ENST00000360184
CBT15_circR_4654	3.76	0.0001098	63789820-63790790	ENST00000245663
CBT15_circR_3816	3.63	0.007080021	47362475-47362693	ENST00000328771
CBT15_circR_1273	3.59	0.000138797	68347846-68348243	ENST00000294304
CBT15_circR_2676	3.49	0.012859288	4466152-4469465	ENST00000283429
CBT15_circR_2864	3.40	0.012474985	89098590-89102759	ENST00000317447
CBT15_circR_5953	3.33	0.000219072	38990948-39003622	ENST00000296782
CBT15_circR_2662	3.20	0.034070486	346147-347106	ENST00000262320

Table S3-3. Top 20 differential expression circRNA of SARS-CoV-2 infection

Running_number	LogFC	Pvalue	Locus	Best transcript
CBT15_circR_2570	-3.44	2.23987E-06	18797924-18798834	ENST00000567078
CBT15_circR_6977	-3.40	3.01581E-06	18799294-18804898	ENST00000286485
CBT15_circR_4556	-3.146	1.94959E-05	3907925-3912634	ENST00000316562
CBT15_circR_5608	-2.89	0.000101897	74174505-74201370	ENST00000325278
CBT15_circR_714	-2.84	0.000143471	11997671-12014184	ENST00000356352
CBT15_circR_4645	-2.81	0.000171291	62905886-62914406	ENST00000266070
CBT15_circR_6617	-2.80	0.002458721	24623662-24668660	ENST00000432190
CBT15_circR_911	-2.76	0.000230186	68788149-68788328	ENST00000265872
CBT15_circR_4136	-2.69	0.004307819	223998114-224001922	ENST00000258405
CBT15_circR_6092	-2.67	0.000378561	94643272-94654752	ENST00000265140
CBT15_circR_323	3.60	6.85732E-07	2302977-2304585	ENST00000378536
CBT15_circR_7576	3.39	3.64453E-06	3597-3789	ENST00000361390
CBT15_circR_1723	3.28	7.97443E-06	70278131-70311017	ENST00000229195
CBT15_circR_7194	2.95	0.001644986	110941420-110973558	ENST00000374430
CBT15_circR_5953	2.90	0.000102672	38990948-39003622	ENST00000296782
CBT15_circR_5988	2.89	0.001915148	58988492-58993465	ENST00000309641
CBT15_circR_6324	2.80	0.004827675	47253255-47286595	ENST00000296861
CBT15_circR_5201	2.79	0.003171312	31576395-31580096	ENST00000295770
CBT15_circR_5965	2.72	0.006261545	43292473-43298975	ENST00000325110
CBT15_circR_3084	2.66	0.007415919	39708320-39710481	ENST00000269571

Table S4-1. Top 20 differential expression miRNA of panH1N1 infection

Running_number	LogFC	Pvalue
hsa-miR-223-3p	5.76	0.002719401
hsa-miR-133a-5p	5.76	0.001197401
hsa-miR-133a-3p	5.56	0.00155117
hsa-miR-133b	5.40	0.003279399
hsa-miR-451a	5.31	2.68739E-08
hsa-miR-4470	5.06	0.005459552
hsa-miR-95-5p	4.78	0.015537353
hsa-miR-3189-5p	4.31	0.019132269
hsa-miR-4688	4.29	0.029102866
hsa-miR-450b-3p	4.27	0.049894132
hsa-miR-302a-5p	-5.02	0.009393562
hsa-miR-3170	-4.81	0.012095588
hsa-miR-4759	-4.27	0.00010251
hsa-miR-7704	-3.89	5.06841E-19
hsa-miR-4508	-3.46	3.70807E-11
hsa-miR-122-5p	-3.34	2.47599E-06
hsa-miR-302b-3p	-3.28	4.5216E-05
hsa-miR-4516	-3.14	0.005314758
hsa-miR-1915-3p	-3.03	0.015642957
hsa-miR-3150a-3p	-3.00	0.046223505

Table S4-2. Top 20 differential expression miRNA of H7N9 infection

Running_number	LogFC	Pvalue
hsa-miR-133a-3p	6.82	5.64939E-06
hsa-miR-133b	6.80	1.07166E-06
hsa-miR-133a-5p	6.77	7.02634E-06
hsa-miR-4470	5.13	0.004638365
hsa-miR-202-5p	4.32	0.019361908
hsa-miR-450b-3p	4.18	0.040332394
hsa-miR-6777-5p	4.16	0.035002985
hsa-miR-887-3p	4.14	0.047613308
hsa-miR-190a-3p	4.06	0.049030713
hsa-miR-3189-5p	4.05	0.042520577
hsa-miR-4516	-5.38	0.000741682
hsa-miR-4508	-4.73	1.52392E-14
hsa-miR-1915-3p	-4.56	0.015649343
hsa-miR-7704	-3.62	9.62166E-18
hsa-miR-302b-3p	-3.27	3.04959E-05
hsa-miR-548ap-5p	-2.83	0.006319475
hsa-miR-6805-5p	-2.75	0.04893301
hsa-miR-6827-5p	-2.43	0.004919505
hsa-miR-122-5p	-2.41	0.00015413
hsa-miR-4797-3p	-2.35	0.000271869

Table S4-3. Top 20 differential expression miRNA of SARS-CoV-2 infection

Running_number	LogFC	Pvalue
hsa-miR-4470	4.06	0.047292463
hsa-miR-3940-5p	3.30	0.007161687
hsa-miR-4733-5p	3.14	0.014630097
hsa-miR-4684-5p	2.81	0.047571789
hsa-miR-676-3p	2.22	0.024546087
hsa-miR-135b-3p	2.04	0.023385746
hsa-miR-4687-3p	1.88	0.043642683
hsa-miR-4759	1.86	0.00067405
hsa-miR-2277-3p	1.85	0.013575979
hsa-miR-4516	1.68	0.003211578
hsa-miR-143-5p	-4.66	0.020672182
hsa-miR-6827-5p	-4.59	0.004097695
hsa-miR-206	-4.25	0.045097741
hsa-miR-548ap-5p	-2.96	0.012770027
hsa-miR-3605-5p	-2.39	0.04516632
hsa-miR-143-3p	-2.20	0.00431736
hsa-miR-302b-3p	-2.03	0.014930239
hsa-miR-4797-3p	-2.03	0.014080794
hsa-miR-4684-3p	-1.87	0.047314347
hsa-miR-5697	-1.86	0.038594387

Table S5-1. Top 20 differential expression lncRNA of panH1N1 infection

Running_number	LogFC	Pvalue	Locus
NONHSAG097327.1	5.38	1.9273E-08	7:-:128112587-128112824
NONHSAG097813.1	4.65	3.74267E-07	7:+:38702151-38703318
NONHSAG097538.1	4.62	3.06431E-07	7:+:2670534-2671572
NONHSAG048732.2	4.55	3.33463E-06	7:+:128112042-128118723
NONHSAG099846.1	4.36	5.25414E-07	8:-:143895581-143895801
NONHSAG071826.1	4.24	4.97516E-06	16:+:75136966-75229739
NONHSAG109470.1	4.15	1.32592E-05	19:-:2294742-2298864
NONHSAG061959.1	4.11	3.16619E-05	10:+:33126643-33127435
NONHSAG108586.1	4.09	1.42631E-05	17:+:9895483-9896447
NONHSAG109479.1	3.91	5.61783E-06	19:-:4518953-4519531
NONHSAG109088.1	-4.70	2.41965E-08	18:+:50374996-50383284
ENSG00000236027	-4.68	5.88463E-07	11:+:125788111-125791600
NONHSAG084376.1	-4.36	2.38739E-05	KI270733.1:+:122273-135645
NONHSAG098705.1	-4.33	2.44868E-06	8:+:25470827-25471632
ENSG00000203811	-4.08	2.0052E-07	1:-:149839538-149841193
NONHSAG112126.1	-4.04	1.0386E-05	4:-:112604559-112604813
NONHSAG053901.2	-3.99	0.000577934	GL000220.1:+:97129-156152
NONHSAG063717.1	-3.99	9.76731E-08	11:-:125846168-125860797
ENSG00000250536	-3.96	2.12323E-08	1:-:214605470-214608840
NONHSAG049792.2	-3.95	3.1172E-05	8:+:25504412-25504978

Table S5-2. Top 20 differential expression lncRNA of H7N9 infection

Running_number	LogFC	Pvalue	Locus
NONHSAG076212.1	5.93	0.001604567	19:+:48887932-48891725
ENSG00000240023	5.62	1.80391E-07	14:-:35388121-35389069
NONHSAG090683.2	5.60	5.37908E-07	5:+:171494734-171502854
NONHSAG073924.1	5.51	1.48491E-05	17:+:1325377-1326270
ENSG00000134321	5.45	1.045E-06	2:+:6865806-6898239
ENSG00000204252	5.44	5.13171E-06	6:-:33004178-33009612
ENSG00000169327	5.43	1.19349E-06	14:-:21148370-21159060
NONHSAG018621.3	5.39	0.000861138	16:-:11818293-11828845
ENSG00000182393	5.07	3.86654E-06	19:+:39296325-39298673
NONHSAG085240.1	5.05	3.04629E-07	3:+:185890459-185892642
NONHSAG049792.2	-4.88	1.08132E-05	8:+:25504412-25504978
NONHSAG098705.1	-4.87	2.0475E-05	8:+:25470827-25471632
ENSG00000163009	-4.61	0.000238262	2:+:10141382-10211725
NONHSAG023735.2	-4.58	3.08649E-05	18:+:39647396-39649594
NONHSAG073285.1	-4.52	6.38362E-06	17:+:61849872-61850104
ENSG00000212432	-4.49	7.58154E-06	12:+:9445058-9445205
NONHSAG037014.2	-4.49	6.65993E-06	3:+:193996877-193999474
NONHSAG111936.1	-4.48	1.95545E-05	4:+:177309837-177319095
NONHSAG109708.1	-4.46	0.000251946	2:+:10141382-10211725
NONHSAG029753.2	-4.34	0.00083981	2:+:168860204-168864856

Table S5-3. Top 20 differential expression lncRNA of SARS-CoV-2 infection

Running_number	LogFC	Pvalue	Locus
NONHSAG105679.1	3.20	0.000249802	1:-:219247401-219247863
ENSG00000199482	3.09	0.000327814	16:+:10496272-10496374
NONHSAG024482.2	3.06	0.000278631	19:-:3544183-3557569
NONHSAG111319.1	3.05	0.000288668	3:+:126476241-126477394
NONHSAG041097.2	3.03	0.000710579	5:-:97223371-97227957
NONHSAG003647.2	3.02	0.000475728	1:+:181236388-181238604
NONHSAG081469.1	2.98	0.000422962	20:+:32564992-32608893
NONHSAG090209.1	2.98	0.000381387	5:+:61198337-61217927
NONHSAG096636.1	2.95	0.000667622	7:+:125043037-125045026
NONHSAG036027.2	2.93	0.000802537	3:-:128843564-128844404
NONHSAG080416.1	-3.23	8.11765E-05	2:+:121771189-121772218
NONHSAG109525.1	-3.16	0.000115407	19:-:18122129-18123280
ENSG00000236675	-3.16	2.26469E-06	1:+:155230975-155234325
NONHSAG111399.1	-3.05	0.000153195	3:+:183687274-183687959
NONHSAG105222.1	-2.95	0.000580575	1:+:235432985-235433231
NONHSAG055783.3	-2.93	0.000561968	Y:-:12661090-12662233
NONHSAG109911.1	-2.89	0.000545935	2:+:132347522-132347846
NONHSAG106486.1	-2.88	0.000652249	11:-:46594322-46595445
NONHSAG014134.2	-2.88	0.000585138	13:-:106557048-106562044
NONHSAG023646.2	-2.87	0.000435489	18:+:31884858-31885143

Table S6. The sequence of LncRNA34087.27, IRF1-3'UTR and miR-302b-3p.

LncRNA34087.27 (Chromosome 14:+:73851844-73932511)

CATCCCTCCTGGATCTGGCGGGCCCGGGAGCGGCCAGTCTGAAGCCCTGCGGCTTTGG
CCCTTCCCCTCGCGGGTTTTAGGGGCGATGTTCTTGCTCCTTTGCTTGGTTTTGGGGG
TAGGGATATAGGAACAGACTGTCCTGAGAAAGCCAGAGTCAGCGAGTCCCCGGGGCG
GGGGCTCTTGGACTGCACCCGCAGCCGGGGCCTGCCTACGGACCTTGGGGCCTTATTT
CTGAGGCCTTTGTGGTGTGGGGTAGAGGATGCCAGGAGGCCTTGCCCTGAATGTTTCA
GTCCAGAGCTACCCTCTCCTTTCATACCACTTACTTTCTGGGTTTGGAAAAGGGACAGC
TGCCTACTCGGCTGGGCTGGGTCGCCCGTTTCTCCTTTGCCTGTCAGTTCGGGGGGCA
GGAGTGTGTGGATTGTTTACGGCCTTCATACATTTCGTGTCGAATTTATTTTCTGAAGTT
TCTCAAGATAATCCAATTTATTAAAGTTTATCCCACTTGATCATGAGGCTCAGAAATGCA
TTTAGGCACCATTTGGAAGTTAAATATTATTAACTTGGGACTTAAGAATTAGTACTTAGG
TTGGAACGAAGGTCAACGCAGCATATTTGCAATAAATAAAAAACATTTGGAAATAAAG
TCGGGCATTTTGTTAGGGAGTGAGAAATTGCACTTGGTTTTTTCTTAGTGTGGTGCAGA
GTAAAAGACAACTAAAGTAGCAGTCAGAAGTTTTAAGTTCTAGTTTTTTACGTTTTGCA
TCTAAGTAGATGTCTAATTTTTGGCAAGTTGCTGAATCTCTAGCTCTGGGGAGTTTTTCT
GTAAACGAAGGGGTTTACCTTTAGGGCTATGACAATTTTTCCGAGGCACTGAACTTTGA
GTAGAAGTGACATAAGCATTTCTTTGTTTTGGAAAGATAACTTGAATGGCTGCATAAAG
GATGTATTGGAAAGAGGAATTTTAAACGCGGGGTGACCAGTTAGAGGCCTGTTTTAATA
GTATAGTAGAAAGTTACTGAGAGACCTAAACAGGGCATTACAGTAGGGAACAGAGAAC
ATTCTGGGAACAGAGAATCACGGGAATTATTTAAAAATGGAGAATGAGAGAGATTTAA
AGACCTAGAATTTGCAGCTGAGTGGGAGGAAGATTGGGCGATCATTGAAATAGGTAAT
ACAAGAAGAACAGGTTTGGTAAGGAAGATAATAATCTGGATTTTGGACATTGTTAGGTT
TGAGATGCTGTTGGATATACATGTGGAGATACCTAGCAAGCAGTTAGAAATGAAGGTTT
GGAGTTCAGAAGAGATGACTAGAGAAAAATGTAGGTATCATTTGTAGTGTCATTCATTC
ATTATACAAATATTTAGTACCTACCAGTTGCTTACTGGGAAGCTTGTATGGAAGCCCTTT
AAGCTCATAGTAAACACATTAGAAGTGGGAAGTGACCGTTAATGGGCACAGGGTTTCC
TTGTGAGGTGATGAAATATTGTAAAATTGATCGTGGTTCTGCTTGTACAACTCTGAATG
AACTAAAGATATTTTAATTGTACTCTTTAAATGAGTCAATAGTATGGTATGTGAACGATAT
CTTAGTGAAACTAATTTTTAAAAAAGATTTACATTGCAGTTTTGGAAATCTGTTGAGTA
AGGAATTGGGAATAGGAATGTTTAATTGTGTATTTGCCGTTACACCTATAGCTAATGTAC
ATAGTACGTTGCTCTGTTAAAGATGAGATCCCCACAGTACACTTGATAAGATACTGAGT
GCTCTAGTACAAATTCTTTTGAGTTCAGTTGCATATCTTAATGCATCCTGCTATTTTTTAT
TTTATTCATACCGTACACATACAAATCATAAACTTGGACACAAAAATTGTATGGTAGTAC
TTAGTTATAATATGAATACCCAGTGCTTGAGAAAATGCTCGTGTTAATTTTTAAAGATTAT
TGAAGTGAAAACACTGAAATAAAAAGATGTCATAGATAACATAAAAAGGACAGTAAAT
AGTTGTCTGGTTCCTCAGAGATTTTACAATCCTGGAATCTCAAAGTTGGAGGAACCTTA
AGGATGACTTAAACAACAAACATCTGATAATTTCTTGGCTTGTTAAGACAAGCTGGAAT
TGGGGAAGAGGAATTCTTGTTTTTTTTGAGACAGCCTCCATTTGTTACCCAGGCTGGAG
TGCAGTGGCGTGATCACTGTTCAGTGCAGCCTCGATCTCCTGGGCTAAAGCAATCCTCC

TACTCAGCCTCCCTAGTAGCTAGGACCACAGGCATGTGCCACCATGTGATGCTAATTGA
AAAATTTTTTTTTGTAGAAATAGAGTCTCACTGGCTGAGATCGCGCCACTGCACTCCAG
CCTGCGTGACAGAGCGAGACTCAGTCTCAAAAAAAAAAAAAAAAAAAAAAAAGAAATA
GGGTCTCACTGTGTTGCCTAGGCTGAGAAAAGGAATTCTTAGGCCATGGAGATTCAGG
GTCAAACCTAGAGAGATGTAATCACTATTCACGCCAATGATTTCTGCATACTTCTGGTGT
GGGTGGGAGTCCTGGCTTTGCAACTTAATAACTTGGTAATTAAGTAATATACTGAGCCT
TGGTTCCTTCCTCTCTAAATAGAGGGATGGATAAACCATATGAAACTGTTGCAGTGCTT
GAATAAGAAAAATATGTGTGGAAGAAAAGTCAGGGATTTATGATACTTTATGTGGGAAA
CTTGGAAGGAGCATTTCCATTTATATCTTGTAAATGGTTAGTTTTTTTTTTTTTTTTCTCG
ACACAGAGTCTTGCTCTGTCGCCCGGGCTGGAGTACAGTGGAGCGATCTCGGCTCACT
GCAATCTCCATCTCCTAGGTCCAAGCGATTCCCCTACCTCAGCCTCCTGAGTAGCTGGG
TCTGCAGGCACATGCCACCACACCTAGCTAATTTTTTGTATTTTAGTTGAGACGGAGTT
GCACCATGTTGGCCAGGATGGTCTCAATCTCCTGACCTTGTGTTCCGCCTGCCTCGGCC
TCCCAAAATGCTGGGATTACAGGCGTGAGCCACTGCTCCCGGCCTAAATGGTTACTTTT
TTACAATGAAAAATGAGGATTTCAGGTAGCTTAGAAAGAGAGAGGGAGAAACCAACA
GGACAAAAAAACATTTATTCAGTTACTTTTTTTTTTTTTTGAGATGGAGTCTTGCTCTGT
CGCCCAGGCTGGAGTACAGTGACGCAGTCTCAGCTCACTGCAACCTCCATCTCTCGGG
TTCAAGCGATTCTCCTGCCACAGCCTCCCGAGTAGCTGGGATTACAGGCACCCGCCAC
ATGCCCGGCTAATTTTTGTATTTTTAGTAGAGACGGGGTTTCGCCATATTGGCCAGGCTG
GTCTTGAACTCCTGACCTCAGGTGACCCACCCGCCTCGGCCTCCCAAAGTGCTGGGTT
TACAGGCGTGAGCCACCGTGCCCAGCCTAAGTGCCCTCTTTGTAAAGTCTGTCTTGTTC
TACCTTTCCACACTACCTGGTATTAATTGGAAACTTTAAAGTACCCATTGATTGAAAATA
TACCCATCCTGGATACATTTTACACTGTATGATTATTTGTAATAGTGCTCCCAATTTTAGT
AACAATTGTACAAAATAAAATGGACTCACATTCCAAAATCCTGAAAAGAAACTAAGCT
ATAAGAAAGTAAGAATTTAAGTTATAGGGAGTGGCTGGGCGCAGTGGCTCACACCTGT
AATCTCAGCACTTTGGGAGGCCACGGCAGGCGGATCACCTGAAGTTGGGAGTTCGAG
ACCAGCCTGACCAACATGGAGAAACCGTCTCTACTAAAAATACAAAATTAGCCAGGTG
TGGTGTCTCATGCCTGTAATCCCAGCTACTCCAGGAGTCTGAGGCAGAATTGCTTGAAC
CTGGGAGGCAGAGGTTGTGGTGAGCTGAGATCGTGCCATTGTGCTCCAGCCTGGGCA
ACAAGAGCAAAACTCCATCTCTAAATAAATAAATAAAAGAGAGAGAACAGCCGCATCA
GGGTCCTGAGAGCACTACCTTTGGGAAACGCTCTTCAAATTTTAACTTTTTTTTTTTTA
AATGTAATTTTAAAAATTATCAAAACAATATGGTTGCCTTTTAGAAATATTGGAAAATTG
AGAAAAGAAAATTAAGTCACACCAGCCTACCACACGGTATAGCTACGGTCATAGCTGT
ATGAATCTCCTTTTACTTTTTCTGATGCTTATTATAACTAGTTTTAATAATAGCAAGTATAT
AATTTTGTATTTTATTTTTATAATTTAACATGTCAGTCACTTGTTGTTGCATTGTCTTCATA
GCATTTGTTTGTTGGTTTGTTTTTTGAGATAGAGTCTTGCTCTGTCGCCCAGGCTGGAG
TGCAGTGGTCTGACCTTGGCTCACTGCAACCTCCACCTCCTGGGTTCAAGGGATTCTC
CTGCCTCAGTCTCCCGAGTAGCTGGGATTACAGGTGCCTGCGACCATGCTCAGCTAATT
TTTGTATTTTTAGTAGAGAGGGGGTTTCACTATGTTGGCCAGGCTGGTCTCGAACTATT
GACCTCAAGTGATCCACCTGCTTCAGCCTATCAAAGTGCTGGGATTACAGTTGTAAGCC
ACCACACCCAACCCTTCACAGCATATTTTGAAGTGACAGTGCCATATTTAATCAACCAT

TCCCTAATTGGGGGTGTGTGTGTGTGTATACATTGCTTCCAGTTGTTTATTACATAGGAT
AAGACGGACATCTTTGGACATAGCTCTATCCATATTTAAGATAGTTTTTTAGATTATTAGA
GGTTGAAATACTGCATCAAAGCGTAGTATTTTTATGGGTTTTGATGCCTGTTGAGCTTGT
TGTATTTTAAAAGAAGTGTTTCGGTTTGAATTCTTTATTTGCTTGCTTGTTGTGATTTTTT
TTGTATATGTAAAGAAGTGTCTTTATGTACCTGTGTTTTCCCACTGCTCTTAATGCCCCC
TCTCTCTTTTTTACCCCTATTCTAGGTTACATTGATTACCCACCTAGTACAACATCTTACG
GGAAGAGCATAGTATTTCCTAGAGGAATATGAACATAACAGGAAGGTATCATTGGCTCT
GAATTAAATTTGAACTTGTCCCCTGAATAGCTACAGGTTTTGGAAGCTGAATCAATGTT
ATCAGATGAGTTAGAATCCAAACCAGAGGTGAGCCCAGTCAGCTTGTTTCCTTTTCTTT
TGGTGGGCTATATTTCAAAGTTTATCTTCAGTTTAGGGGGTCAGCAAACTTTTTCTTTAA
TGGGCCAGATAATAAATATTTTAGGTTTTTTAAGCTATATGGTTTTGATTGTTACTATTTTT
TCAAAGATTTAAAAATTATATTTTTTGTGATTGTTTTTATATCTGTTTTTCAGTATATGTGA
AATCATACATTTAGATAATGTAATAAAATCTTATAAATAAAAATATATGTTTATGTATAAAT
ATATACATAAATATGAGATACATGTGATGTATAGGTGTATAAGTATATAAATATTTACTACT
ACAAATTCTTTTGTTTATTTTATATATATCTGTAAGTATTTTCTTTCTAAATGTGATTGGGG
ATTTAAACATTTTTTGTTGTTCACTTTTCTTACTTTAAATTTAGCACATCTGTGTTGCAGA
GTGGTCACTGAATTTGGAATCAGATTACTTAAATTTGAGGATTGGCATTGCTCCTAACTA
GTCTTGTGACCTTAGACAAGTCACTTAATGTCTTCAATTTTATTCCTCAGCTGTAAAATA
TGCCTTAAGTTTGGGTGCGGTGGCTCACGCCTGTAATCCCAGCACTTTGGGAGGCCGA
GGCAGGTGGATCATGAGGTCAGGAGTTCAAGACCATCCTGCCTAACATGGTGAAACCC
TGTCTCTACTAAAAATACAAAAAAAATTAGCTGGGCGTGGTGGCGGGCACCTGTAGTC
CCAGCTACTTGGGAGGCTGAGGCAGGAGAATGGCGTGAACCCAGGAGGTGGAGCTTG
CAGTGAGCCAAGATCATGCCACTGCACTCCAGCCTGGGCAACAGAGCAAGACTCTGT
CTCAAAAAAAAATACATCTTAAATCTTACTGGGTTAATGCGATAATTAAATGAGCCAGT
ACACAGAAAAGTGTTTTGTAAAATGTAATCAATATGATGCAGTTGATTTTTTTTAAGCC
ATTATTTTAAAATGTAATTATTTTTATCATTTCTCTGTTTGACAGAAGGTGGCAGAGGTTA
ACTTAATTACAGTGAAGTGTCACTTAGCGATAGAGATACATTCTGAGAAATATGATGGT
AGTTGATATCATTATTATGCGAACATGGTAGAGTATACTTACACAAACCTGTATGGTTTA
GCCTGCTACACTCTAGGCTGTATGGTATACCTTATTGCCCCTAGGCTGCAAACCTATACA
GCATGTTACTGTACTGAATACTGTAGGCAGTTGAAGCACAGTGGTAAACATTGTGTATC
TAAACATATCTAAACGTGGAAAAAGTACAATAAAAATAGGATATTACAATCTCACAGGA
CCATCATCCTATATGTGGTCTGTTAACTGAAATGTCGTTTTGCAGCACATGACTTTATATA
AATCAGCTGTTCTCTAGAACAGAGAACTTGATAAAATAACTTTAGATATTATGTTGTTTA
GTTTTTGTTTGAATATGGATGAGACTAGTGAACAGTCTTAATATTATCTTTTGGTAAATTC
AAAGGTTTAGATACATTTCTAACAACTCGTATTTCTCAGTGTTGTCTTTTCTCCCCCCAG
CTCCTGGTACAGTTTGTTCAGAATACGTCCATCCCATTGGGACAGGGGCTTGTAGAATC
AGAAGCTAAAGATATTACTTGCTTGTCCCTCCTTCCCGTGACTGAAGCCTCAGAATGCA
GTCGGCTAATGTTACCAGGTAAAAATTAAGATCACTAGAAATAGGATAAAGAAGTCTTA
AGAGCTTAGCCTACTCTATGTTACTGGTGGAATGAATATATCGTTTAATAACTGACTGGT
GGAAACTGTAAAAATTAGGAGTCATGGAGAAAAAGCAACCATGAAATCATTAGTGCAT
TGGTAGTTTAAGCCATAGGAATGGATATGATCACCCAGAGAGTGTGAGAGTGGGGAAA

AAAGGTCAGTGCAAAACTATGGAACTCTTTATTTAAGGTACAGGGTAGGGCAGGAGAA
CTTGTAAAGGAGGGAAGAAACAAAGACTAAAATAGTTGGATGAACATCAGCAGAGTG
GAGGCATGAAACCCAGGAGAAATCATTGTATCAAATGCTACTGACAGGCCCAGTGAAA
TAGGGACTAAAAGGACTCCATTATTGACTTTGGCAGAACCTGTTCCATGGAGAGAAGA
GGATGGAAAGTCAGGCTGCAGTGACTTGAGTATACATACTTGCTTTTTTTTTTTTTTTG
AGATGGAGTGTCACTCTGTCGCCCAGGCTGGAGTGTAGTGACGCAATCTTGGCTCACT
GCAACCTCCGCCTCCCCTCCCAGGTTCAAGCTATTCTCCTGCCTCAGCCTCCTAAGTAG
CTGGGATTACAGGTGCGTGCCACTATGCCTGGCTAATTTTTGCATTTTTAGTAGAAACG
GGGTTTCACCATGTTGGCCAGGCTGGTCTTGAACTCCTGACCTCCAGTGATCAGTCCG
CCTTGGCCTCCCAAAGTGCTGAGATTACAGGCGTGAGCCACTGCGCCAGGCCTATCAA
TACATACTTTCTAAAGTCTCTCCTGGTACGAGAAGGAAAAAGAGAAGGCGGTAAGATA
GAGGGAAATTTTTTGTTTGTGTTAAGATCTGAGATATTTGAGGGTTGCTTTATGCTAAGG
GGAAAGAACCAGTTAGAAAAGTTGAAGACAGGCCAGGTGTGGTGGCTCGTGCCTGTA
ATCCCAGCACATTGGGAGGCTGAGGCAGGAGGATAGCTTGAGCCCAGGAGATTGAGA
CCAGCCTGGGCAACATGGCGAGACCTCGTCTCTACAAAAAATTAAAAATTAGCTGGGC
ATGGTGGTGTGCAGCTGTAGTCCCAGCTGAGGTGGGAAGATCACTTGAGCCCAGGAGT
TTGAGGCTGCAGTAAGCTGTGATCACACCACTGTACTCCATCCTGGGTGGATAGAGCA
ACACCCTGTCTAAAAGAAATAGTAATAAATTTAAAAAATATATGTATAAACAAACAATA
ATTAAGGAAATGAAAACAACAGCGTTCATCTTCCCTTAGAATGACTGTTATCTTAAAGA
TGGAAGATAACAAGTGTTGGTAAGGATATGGAGAAAAGGGAACCTTTGTACACTGTTG
GTGGGAATGTAAGTTAGTACAGCCATTATGGAAACCGTATGGAAGTTCCTCAGAAAAC
TAAAAATGGAGCCACTATATGACCCAGCAATCTCACTTGTAGGTATATATCCAAAGGAAT
TGAAATCAGTAAGTCAAAGATGTCTATACTTACATGTTAATTGCAGCATTATTCACAATA
ACCAAGATATGGAATCAGCTTTAGTGTTCATCAACTAATGAATGCATTTAAAAAAAAAT
GCATTCACCTTCTTTTCCACATTTCTTTTAGTTTTAATTTTGATTTAAAGTATTTTAGACA
TACAAGTATAGATAAAAGTAAATGAAAATCCAAATACTTGGCCTCCCAGCTCATAAAAC
ACAATATTACAGGCCGGGCGTGGTGGCTCATGCCTATAATCCCAGCACTTTGGGAGGCC
GAGGTGGGTGGATCACTTAAGGTCAGGAGTTTGAGACCAGCCTGGCTGGTGAAACCC
TCTCTACTAAAAATGCAAAAGTTAACCAGGTGTGGTGGCACAGAAAAAATAACCGCAG
CCTGAGATACAACCATAGGTAAAAAGGTAATGGCAAAAACTGCAATAGCTTTTGCACC
AACCTAATACAAAAGTTAAGGAGAGGGAGCTCTCTCCAGGAAAAATAAAATGTAGATC
CTATTCTTGAGTTAGTATTCATTGTATAGCTATATTTTTCAAGGAGAAAGTACTGATTTCT
GGCCTCAAAAAGGACTAAATTCTGTGATGTCCAAATGTTGAATGGTTGGTTATTCACCA
ACCATTACTGCCAAGTGACAGAGAATTCTGTTCCTCACGTTTTGGCTGACTGCCTTTGG
AACATAAAGCCATCTCTCTGTTCTTCTGTGAGACAAAGTTTTTATTTGTGTAATGAGAAT
GCTGTCTGTTGCTATAAGGATGTTAGCTTCCAAGAGATGTTGGGTGCTGTGTATCAAAA
TGGTGGGCTCCTAGAGATAGGTGCTACATAAAGCTGTGGTATTTTCCCTTTACTAGATGA
TACTACAAATCATTCTAACTCCTCCAAGGAGGTCCCTTCCTCAGCTGTTTTGAGAAGCC
TTCGGGTGAATGTGGGTCCAGACGGAGAGGAGACGAGAGCTCAGACTGTACAGAAAT
CCCCGGAGTTTTTGTCCACTTCAGAGTCTTCTAGCTTGTTGCAAGATCTACAGCCAAGT
GATAGCACTTCTTTTATTCTTCTTAACCTAACAAGAGCAGGTATTCTTTCCTTTTTTTTGG

GCTCTGCTTATGCCTAAGAAAAAAATTAGGGGTGGGGCATATTTAACTTAGTCATACTTA
AAATAGAAAGAACCTTTATTATTTTATTATTTTGGAGAATTGGTGTGTCTGGTGACCATT
TGAATATGTAGAGGGAGAGGGAGGACTCTAGTATGACTCATATCTTGTTTGGGAAATTA
TGTAGTGCCATTTACTGAGATCGCACAGGAAGAGCAAGGCTTGGGGGAGGATGATTAG
TTCCAATTTGCACCTATTTATGTTGAGATTTTGGAGGTATATTCAGGAGGACATGTCCTT
TAGAGAGTTAGAAACATGAATCCAGAGTTCTAGAAAGGTCTGTTTTGGTGCTTATCAGC
ATTCAGGAAACGTATGAAACAATGAACATGGATTCTAGAGAAGTCTCAAGCATAAACA
GAAAAGAAGGCAAGTCTGGGACCCTGGGAAAAAACATTTAAGAGGACAACAGAACA
GGAAGGAGCAGCTGGAAGGGGATGAAGAGAACTGGGAGAGAGTGGTAGCAGGGAAG
CCAAGGGAGGAAGAAGTTTTAAGGCAGAAAAGATACTAAATCATGCCAAATACTACAT
GATGGGCTTATTAGTGAGAAGAAGTGTGTGTCTGGTGACCCTAGCAAGAGCAATTGTT
ACTGTGGTAGCTGAGGTAGAGGCCAGGGTTAAAGAGATGGGGAGTGGATGGGAGGTA
AGACAATAGATACACAAGTTAGAAGGTACACTTGTTCAAGAAGCTTGACTTTAAAGGA
ACAGGGAAGAAGGTAAATAGCTAGAGAAGTACTTGGGGTTTTCTAATTCTTTTTAAAA
ATAAGGGTGAGAGACTTGAGTTAGGACATATTTAAAGACATAAGGAAATGCTGGCCGA
TAGAGAGGAACTAGTTGCAGATAGGAGAGAGGAGATAACACAAGTATATATGGGGGGA
TAATTGAAGAATGAGAATGAAATACAGTAGGTGGGGATTAGTCTGGTTGACCAAGAAA
GACAGCTTTGCCTTGAAACCAGAGGAGGCAAGGTAATGACAAGCCTGTAGACTAAAG
GTAGAAAGATGATAGCCTGCTCTTCAGTGAGAAGTTTGAGGTAAAAGGCGAAGGTCA
GGAATAGCTTATTTAGGACATGGAGAGTTTCTGGGCCAGGCGCAGTGGCTCACGCCTG
TAATCCCAGTACTTTGGGAGGCCGACGTGGGCGAATCACGAGGTCAGGAGTTCAAGA
CCAGCCTGACCAACATGGTGAAACCCCGTCTCTACTAAAAATACAAAAATTAGCCGGG
CGTGGTGGTGCATGCCTGTAATCCCAGCTACTCAGGAGGCTGAGGCAGGAGAATCACT
TGAACCTGGGAAGCGGAGGTTGCAGTAAGCCGAGATTGCGCCACTGCACTCCAGCCT
GGGTGACAGAGCGAGACGCCGTCTCAAAAAAAAAAAAAAAAAAAAGGGACATGGAGA
GTCTCTGAGCCAGGGCAAATAAAAAGCTTGCTTGGTTTCTAACTTTTCATATATTTTGTT
TCTTCCAGGTCTGGGCTCTTCAGCTGAGCACTTAGTGTTTGTACAGGATGAGGCAGAA
GATTCAGGGAATGATTTCCTCTCCAGTGAGAGCACAGACAGTAGCATTCCATGGTTCCT
CCGGGTTCAGGAGTTGGCCCATGACAGTTTGATTGCTGCTACTCGTGCACAACTGGCA
AAGAATGCAAAAACCAGCAGCAATGGTGAGGCCCGTCGGCATTTTCCTTGCCACTATT
CTGTGCAAAGAGATTTTGGTAATGCAGTGGCTGGTTTGAGTTGTATATAATTTAAATTAT
TCTATTGATGAGAGCAGAAGGCTAGAAATCTGTAAATTGTTTAAATTTTATTTTCTATAA
AATAACTTGGTCATGCCAGATTTGTGTTCAAAACCAACTAGTATTCTGAATTCTCAACTT
TAAATAATGTACAGAGCCCCTTTTAAAGAAAAAATAATATACATCCTGGGGTTTGAATTG
TTTTTATTGTAATGTCTCTTAAATATGCAAAAACATAAATTCTGTGTAAACTTCATATACT
TAGA

IRF1-3’UTR:

CAGGGCCCCTGGGCCCCTCTTATTCCTCTAGGCAAGCAGGACCTGGCATCATGGTGGAT
ATGGTGCAGAGAAGCTGGACTTCTGTGGGCCCCTCAACAGCCAAGTGTGACCCCACT
GCCAAGTGGGGATGGGGCCTCCCTCCTTGGGTCATTGACCTCTCAGGGCCTGGCAGGC

CAGTGTCTGGGTTTTTCTTGTGGTGTAAAGCTGGCCCTGCCTCCTGGGAAGATGAGGT
TCTGAGACCAGTGTATCAGGTCAGGGACTTGGACAGGAGTCAGTGTCTGGCTTTTTCC
TCTGAGCCCAGCTGCCTGGAGAGGGTCTCGCTGTCACTGGCTGGCTCCTAGGGGAACA
GACCAGTGACCCCAGAAAAGCATAACACCAATCCCAGGGCTGGCTCTGCACTAAGAG
AAAATTGCACTAAATGAATCTCGTTCCCAAAGAACTACCCCCTTTTCAGCTGAGCCCTG
GGGACTGTTCCAAAGCCAGTGAAATGTGAAGGAAAGTGGGGTCCTTCGGGGCGATGC
TCCCTCAGCCTCAGAGGAGCTCTACCCTGCTCCCTGCTTTGGCTGAGGGGCTTGGGAA
AAAAACTTGGCACTTTTTCGTGTGGATCTTGCCACATTTCTGATCAGAGGTGTACACTA
ACATTTCCCCCGAGCTCTTGGCCTTTGCATTTATTTATACAGTGCCTTGCTCGGCGCCCA
CCACCCCCTCAAGCCCCAGCAGCCCTCAACAGGCCCAGGGAGGGAAGTGTGAGCGCC
TTGGTATGACTTAAAATTGGAAATGTCATCTAACCATTAAGTCATGTGTGAACACATAA
GGACGTGTGTAAATATGTACATTTGTCTTTTTATAAAAAGTAAATTGTTTATAAGGGGTG
TGGCCTTTTTAGAGAGAAATTTAACTTGTAGATGATTTTACTTTTTATGGAAACACTGAT
GGACTTATTATTGGCATCCCGCCTGAACTTGACTTTGGGGTGAACAGGGACATGCATCT
ATTATAAAATCCTTTCGGCCAGGCGCGGTGGCTCACACCTGTAATCCCAGCACTTTGGG
AGGCCGAGATGGGTGGATCACCTGAGGTCAGGAGTTCGAGACCAGCCTGGTGAAACT
CCATTTCTACTAAAAATGCAAAAATTAGCTGGGCGTGGTTGCGGGTGCTTGTAATCCCA
GCTACTCAGGAGGCTGAGGCAAGAGAATCGCTTGAACCTGGGAGGTGGAGGTTGCAG
TGAGCCGAGAACATGCCATTGCACTCCAGCCCGGGCACCAAAAAAAAAAAAAAAAA
AAAAAACCTTTCATTTGGCCGGGCATGGTGGCTTATGCCTGTAATCCTGGCACTTTGGG
AGGCCAAGGTGGGCAGATCACCTGAGGTCAGGAGTTTGAGACCAGCCTGGCCAACAT
GGTGAAACCTCATCTCTACTAAAAATACAAAAATTAGGCCGGGCACGGTGGCTCACGC
CTGTAATCCCAGCACTTTGGGAGGCAGAGGCGGGCGGATCACGAGGTCAGGAGATCA
AGACCATCCTGGCTAACACGGTGAAACCCCGTCTCTACTAAAAATATAAAAAATTAGCC
GGGCCTAGTGGCGGGTGCCTGTAGTCCCAGCTACTCGGGAGGCTGAGGCAGGAGAAT
GGCATGAACCCCGGAGGCAGAGCTTGCAGTGAGCCGAGATTGCACCACTGCACTACA
GCCTGGGCGACAGAGCGAGACTCCGTCTCAAAAAAAAAAAAAAAAAATTAGCCGGGCC
TGGTGGCGGGCGCCTGTAATCCCAGCTACTGTGGAGGCTGAAGCACAAGAATCACTTG
AACCCGGGAGATGGAGGTTGCAGTGAGCTGAGACTGTGCCACTGCACTCCAGCCTGG
GTGACAAGAGTGAGACTTTGTCTCAAAAAAAAAAAAATCCTTTTGTTTATGTTCACAT
AGACAATGGCAGAAGGAGGGGACATTCCTGTCATAGGAACATGCTTATATAAACATAGT
CACCTGTCCTTGACTATCACCAGGGCTGTCAGTTGATTCTGGGCTCCTGGGGCCCAAG
GAGTGTTAAGTTTTGAGGCATGTGCCATAGGTGATGTGTCCTGCTAACACACAGATGCT
GCTCCAAAAAGTCAGTTGATATGACACAGTCACAGACAGAACAGTCAGCAGCCCAAG
AAAGGTCCTCACGGCTGCTGTGCTGGGTAGCACTTGCCATCCAGTTTCTAGAGTGATG
AAATGCTCTGTCTGTACCGTTCAATACAGTAGGCACTGGCACTAGCCACATGTGCCAGC
TAAGCACTTGAAATGTGGCCAGTGCAATAAGGAATTGAACTTTTAATTGCATTTAATAA
ACTGTATGTAAATAGTCA

miR-302b-3p:

UAAGUGCUUCCAUGUUUUAGUAG
