# Supplementary figures and images for: Competitive Endogenous RNA Network Activates Host Immune Response in SARS-CoV-2-, panH1N1 (A/California/07/2009)-, and H7N9 (A/Shanghai/1/2013)-Infected Cells
Source: Cells. 2022 Jan 30;11(3):487. doi: 10.3390/cells11030487 (PMC8834034; doi:10.3390/cells11030487)

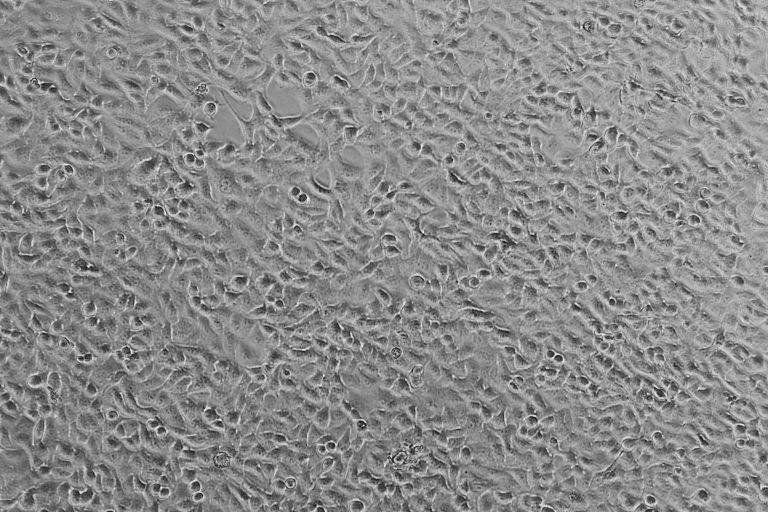

Supplement: Supplementary file 1 [file cells-11-00487-s001.zip › cells-1489030-supplementary/Supplemental Materials/Original gels/24h-C-FigureS1.jpg]

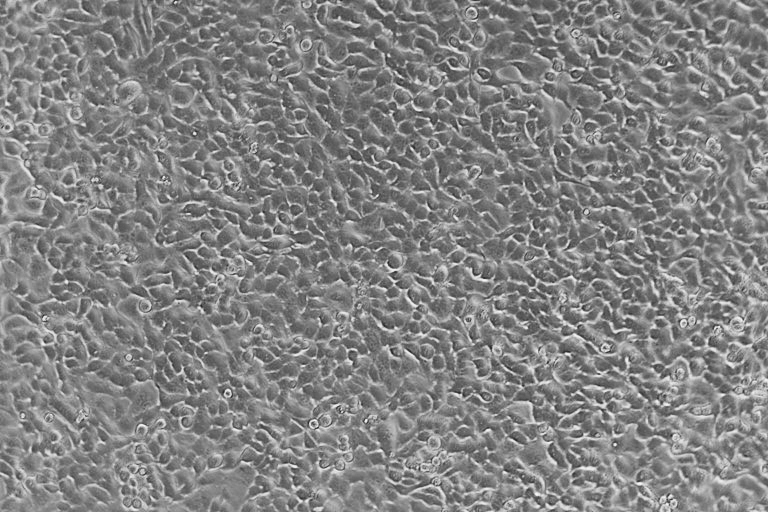

Supplement: Supplementary file 1 [file cells-11-00487-s001.zip › cells-1489030-supplementary/Supplemental Materials/Original gels/24h-S-FigureS1.jpg]

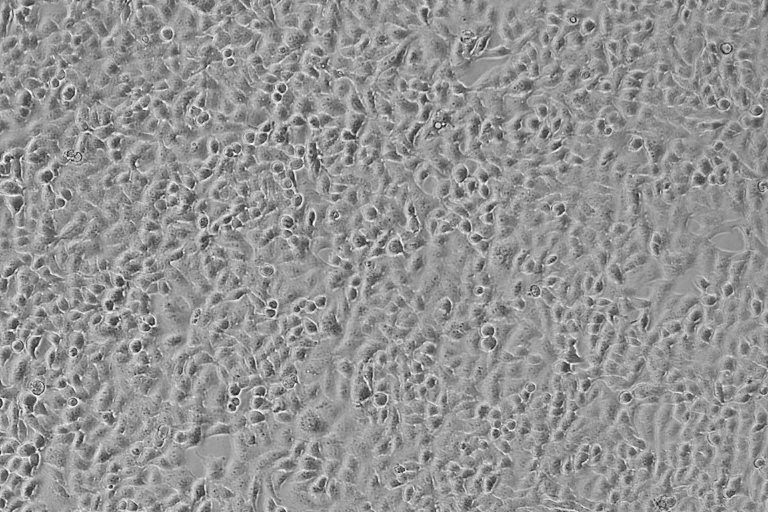

Supplement: Supplementary file 1 [file cells-11-00487-s001.zip › cells-1489030-supplementary/Supplemental Materials/Original gels/48h-C-FigureS1.jpg]

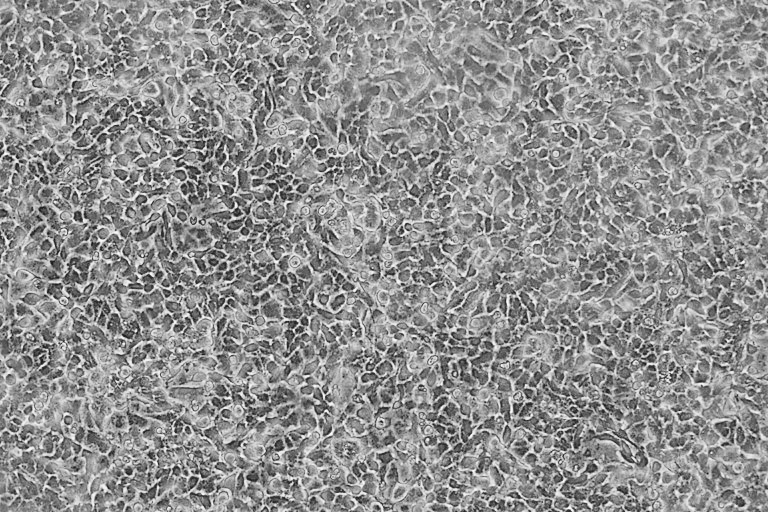

Supplement: Supplementary file 1 [file cells-11-00487-s001.zip › cells-1489030-supplementary/Supplemental Materials/Original gels/48h-S-FigureS1.jpg]

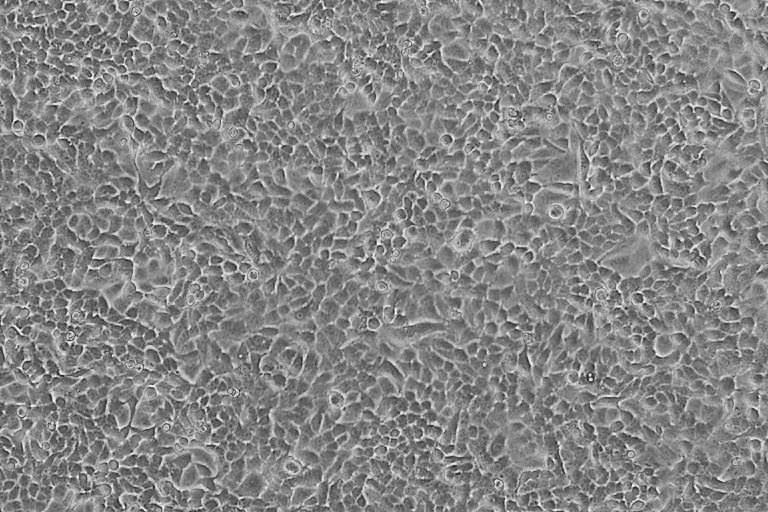

Supplement: Supplementary file 1 [file cells-11-00487-s001.zip › cells-1489030-supplementary/Supplemental Materials/Original gels/72h-C-FigureS1.jpg]

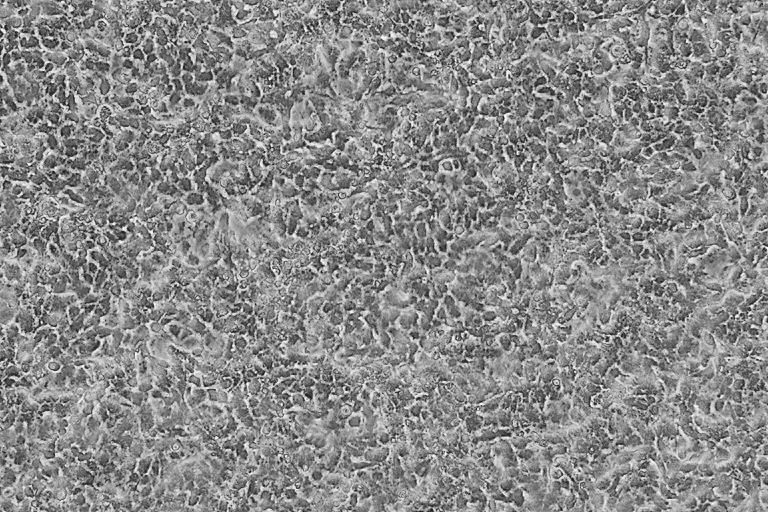

Supplement: Supplementary file 1 [file cells-11-00487-s001.zip › cells-1489030-supplementary/Supplemental Materials/Original gels/72h-S-FigureS1.jpg]

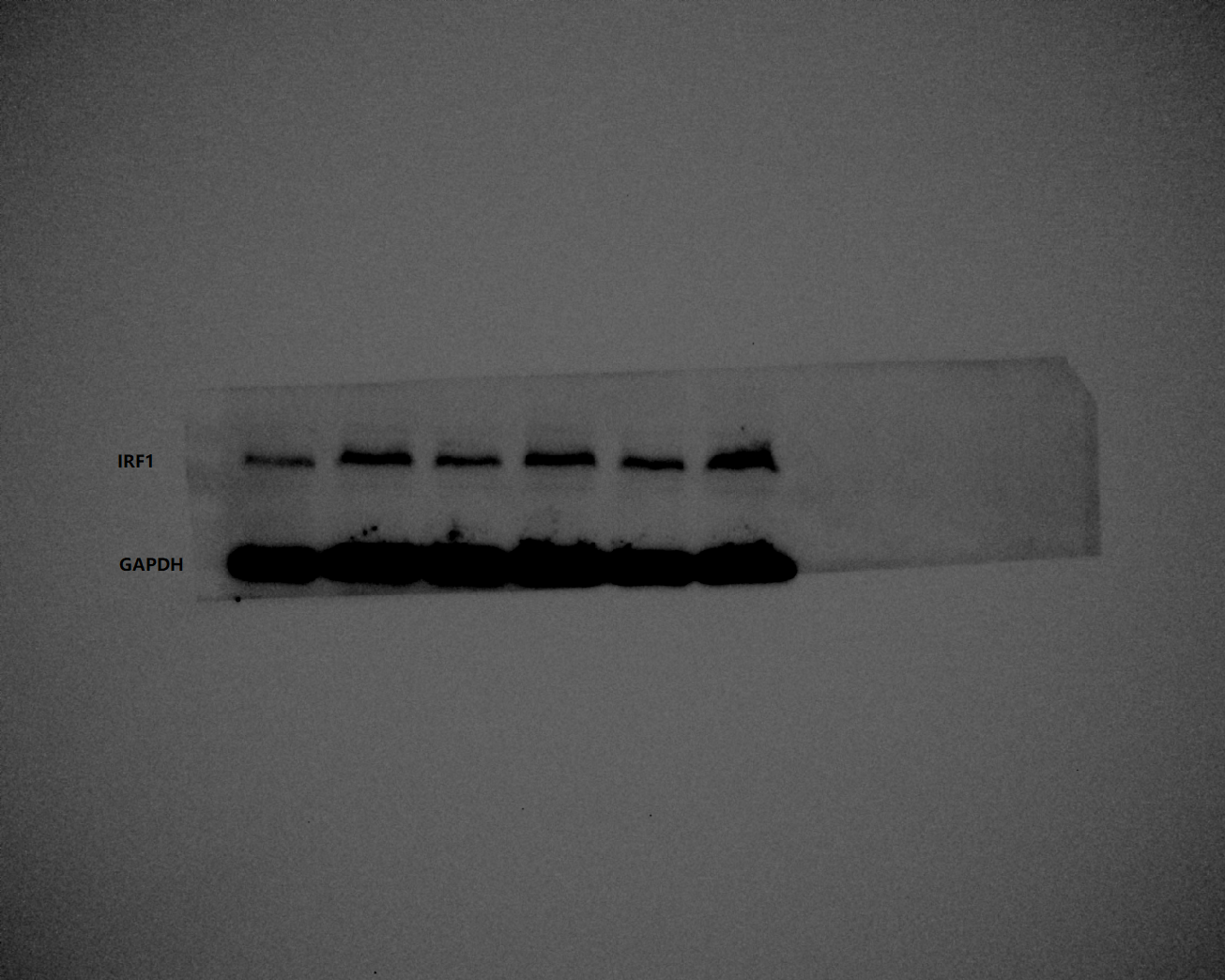

Supplement: Supplementary file 1 [file cells-11-00487-s001.zip › cells-1489030-supplementary/Supplemental Materials/Original gels/FIGURE 6.tif]

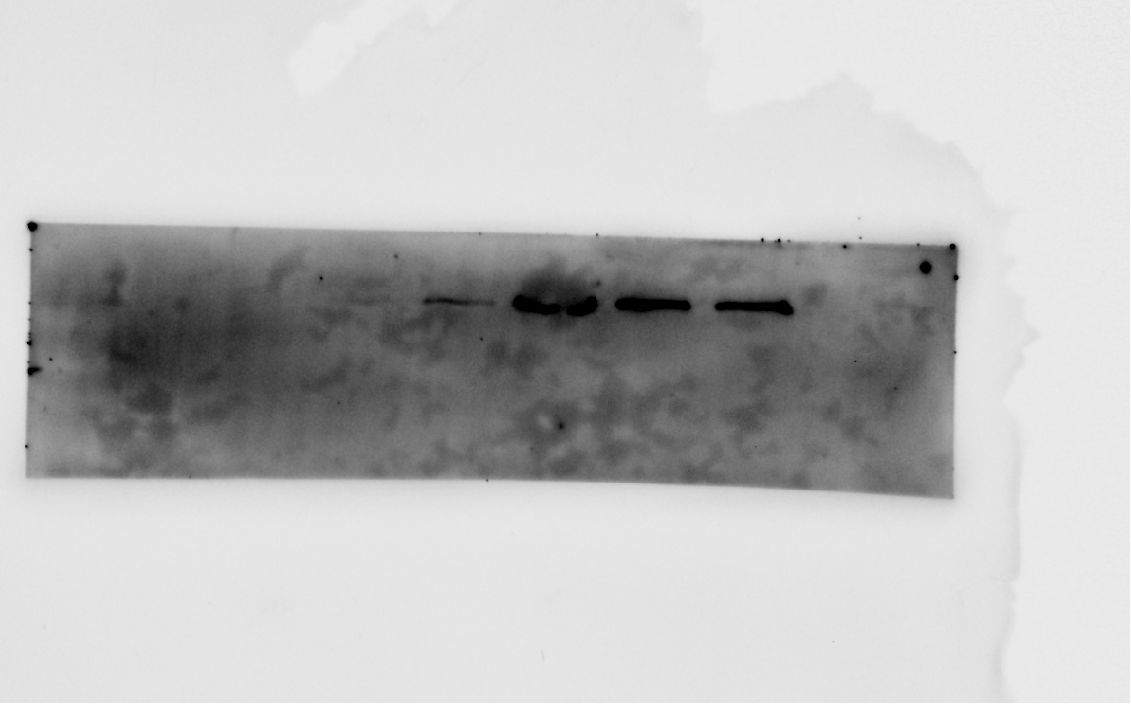

Supplement: Supplementary file 1 [file cells-11-00487-s001.zip › cells-1489030-supplementary/Supplemental Materials/Original gels/FIGURE S1-2.tif]

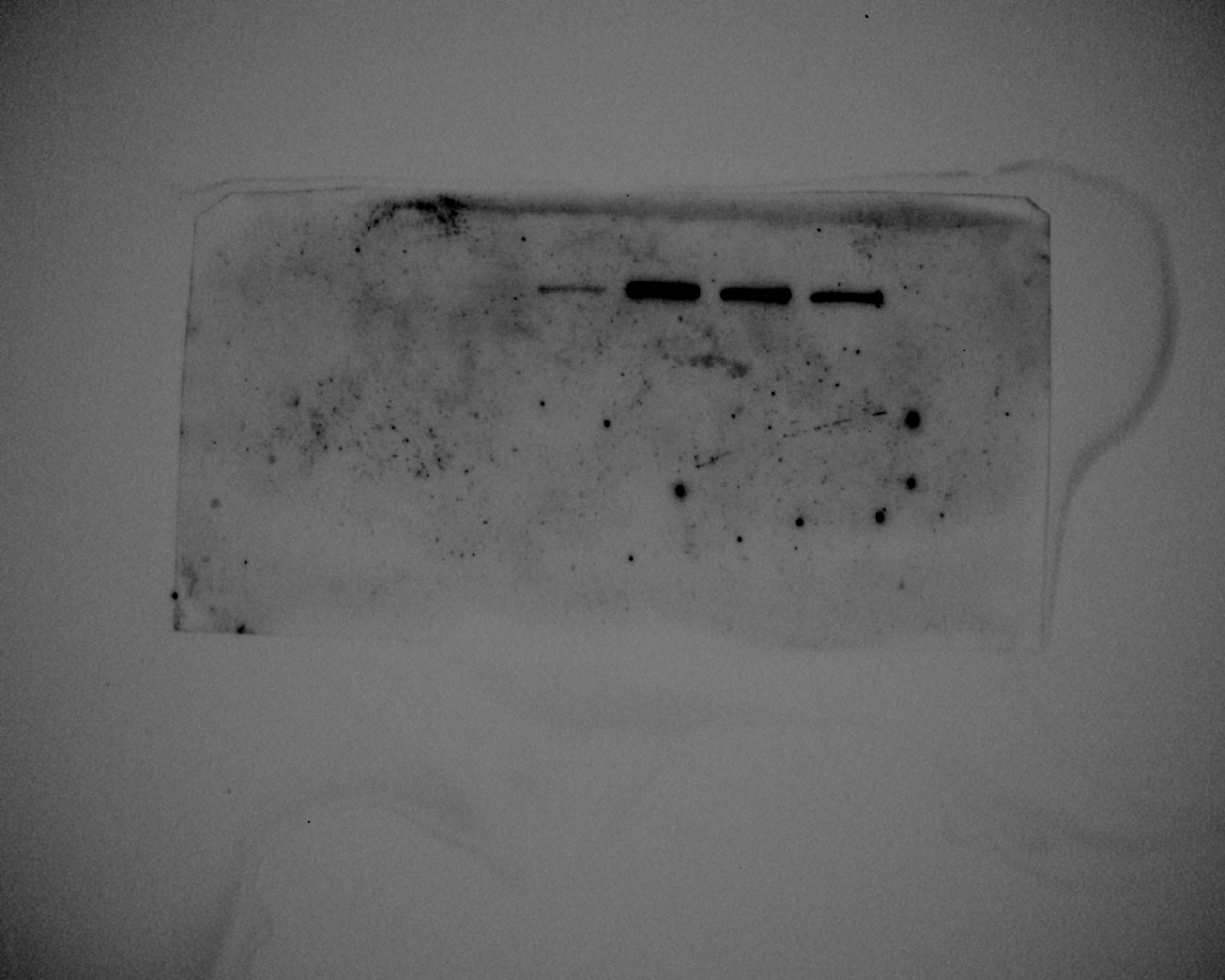

Supplement: Supplementary file 1 [file cells-11-00487-s001.zip › cells-1489030-supplementary/Supplemental Materials/Original gels/FIGURE S1.jpg]
